# A high-quality genome sequence of *Rosa chinensis* to elucidate ornamental traits

**DOI:** 10.1038/s41477-018-0166-1

**Published:** 2018-06-11

**Authors:** L. Hibrand Saint-Oyant, T. Ruttink, L. Hamama, I. Kirov, D. Lakhwani, N. N. Zhou, P. M. Bourke, N. Daccord, L. Leus, D. Schulz, H. Van de Geest, T. Hesselink, K. Van Laere, K. Debray, S. Balzergue, T. Thouroude, A. Chastellier, J. Jeauffre, L. Voisine, S. Gaillard, T. J. A. Borm, P. Arens, R. E. Voorrips, C. Maliepaard, E. Neu, M. Linde, M. C. Le Paslier, A. Bérard, R. Bounon, J. Clotault, N. Choisne, H. Quesneville, K. Kawamura, S. Aubourg, S. Sakr, M. J. M. Smulders, E. Schijlen, E. Bucher, T. Debener, J. De Riek, F. Foucher

**Affiliations:** 1 IRHS, Agrocampus-Ouest, INRA, Université d’Angers, SFR 4207 QuaSaV, Beaucouzé, France; 2 ILVO, Flanders Research Institute for Agriculture, Fisheries and Food, Plant Sciences Unit, Melle, Belgium; 3 Russian State Agrarian University-Moscow Timiryazev Agricultural Academy, Moscow, Russia; 4 Plant Breeding, Wageningen University & Research, Wageningen, The Netherlands; 5 Leibniz Universität, Hannover, Germany; 6 Wageningen University & Research, Business Unit Bioscience, Wageningen, The Netherlands; 7 INRA, US 1279 EPGV, Université Paris-Saclay, Evry, France; 8 URGI, INRA, Université Paris-Saclay, Versailles, France; 9 Osaka Institute of Technology, Osaka, Japan

**Keywords:** Agriculture, Genetics, Agricultural genetics, Genetic association study, Genomics

## Abstract

Rose is the world’s most important ornamental plant, with economic, cultural and symbolic value. Roses are cultivated worldwide and sold as garden roses, cut flowers and potted plants. Roses are outbred and can have various ploidy levels. Our objectives were to develop a high-quality reference genome sequence for the genus *Rosa* by sequencing a doubled haploid, combining long and short reads, and anchoring to a high-density genetic map, and to study the genome structure and genetic basis of major ornamental traits. We produced a doubled haploid rose line (‘HapOB’) from *Rosa chinensis* ‘Old Blush’ and generated a rose genome assembly anchored to seven pseudo-chromosomes (512 Mb with N50 of 3.4 Mb and 564 contigs). The length of 512 Mb represents 90.1–96.1% of the estimated haploid genome size of rose. Of the assembly, 95% is contained in only 196 contigs. The anchoring was validated using high-density diploid and tetraploid genetic maps. We delineated hallmark chromosomal features, including the pericentromeric regions, through annotation of transposable element families and positioned centromeric repeats using fluorescent in situ hybridization. The rose genome displays extensive synteny with the *Fragaria vesca* genome, and we delineated only two major rearrangements. Genetic diversity was analysed using resequencing data of seven diploid and one tetraploid *Rosa* species selected from various sections of the genus. Combining genetic and genomic approaches, we identified potential genetic regulators of key ornamental traits, including prickle density and the number of flower petals. A rose *APETALA2/TOE* homologue is proposed to be the major regulator of petal number in rose. This reference sequence is an important resource for studying polyploidization, meiosis and developmental processes, as we demonstrated for flower and prickle development. It will also accelerate breeding through the development of molecular markers linked to traits, the identification of the genes underlying them and the exploitation of synteny across Rosaceae.

## Abstract (web summary)

A high-quality reference genome of rose is generated by sequencing a doubled haploid line. The researchers analysed the genome structure and uncovered candidate genes associated with major ornamental traits through integrated genetic and genomic analyses.

## Main

Rose is the queen of flowers, holding great symbolic and cultural value. Roses appeared as decoration on 5,000-year-old Asian pottery^1^, and Romans cultivated roses for their flowers and essential oil^2^. Today, no ornamental plants have greater economic importance than roses. They are cultivated worldwide and are sold as garden plants, in pots or as cut flowers, the latter accounting for approximately 30% of the market. Roses are also used for scent production and for culinary purposes^3^.

Despite their genetic complexity and lack of biotechnological resources, rose represents a model for ornamental plant species, allowing the investigation of traits such as bloom seasonality or flower morphology. Furthermore, rose displays a range of unique features as a result of its complex evolutionary and breeding history, including interspecific hybridization events and polyploidization^4–6^. Roses belong to the genus *Rosa* (Rosoideae, Rosaceae), which contains more than 150 species^7^ of varying ploidy levels, ranging from 2n = 2× to 10×^8,9^. Many modern roses are tetraploid and can be genetically classified as ‘segmental’ allopolyploids (a mixture between allopolyploidy and autopolyploidy)^10^, whereas dog-roses display unequal meiosis to maintain pentaploidy^11,12^. Rose breeding has a long and generally unresolved history in Europe and Asia, most likely involving several interspecific hybridization events. Importantly, many very-old varieties are still maintained in private and public rose gardens and are a living historical archive of rose breeding and selection^13^. Large and well-documented herbarium collections, combined with genomic advances, offer excellent opportunities to reconstruct phylogenetic relationships within the species.

Roses have been subject to selection for several traits that are not usually encountered in other crops. In particular, aesthetic criteria have been a principal focus of rose breeding over the past 250 years, next to plant vigour and resistances to biotic and abiotic stresses. Among the aesthetic traits, flower colour and architecture (from 5-petalled ‘simple’ flowers to 100-petalled ‘double’ flowers), floral scent and prickle formation on the stem and leaves have been the main targets of the breeders’ eyes (and noses). Although these traits can be interpreted as signs of the domestication process, they originally evolved through adaptation to natural conditions. The availability of a high-quality reference genome sequence is key to unravelling the genetic basis underlying these evolutionary and developmental processes that accelerate future genetic, genomic, transcriptomic and epigenetic analyses. Recently, a draft reference genome sequence of *Rosa multiflora* has been published^14^. Although completeness measures suggest that the assembly is fairly complete in terms of the gene space covered, it is also highly fragmented (83,189 scaffolds, N50 of 90 kb).

**Fig. 1:**
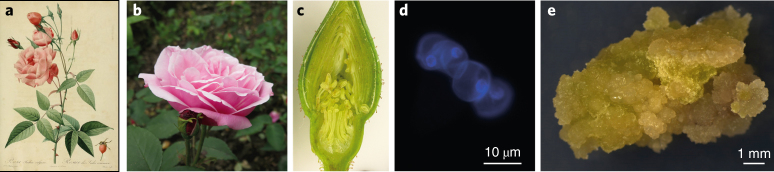
Development of the HapOB haploid line from *R.* *chinensis* ‘Old Blush’. **a**, The *R.* *chinensis* variety ‘Old Blush’ painted by Redouté in 1817. Paul Fearn/Alamy Stock Photo. **b**, A flower from the *R.* *chinensis* variety ‘Old Blush’. **c**, A cross-section of the floral stage used for the anther culture. **d**, DAPI staining on mid-to-late uninucleate microspores. Similar results were observed on more than 15 microspores in one experiment. **e**, The HapOB callus was obtained after the anther culture at the appropriate stage and used for genome sequencing.

Here, we present an annotated high-quality reference genome sequence for the *Rosa* genus using a haploid rose line derived from an old Chinese *Rosa chinensis* variety ‘Old Blush’ (Fig. 1a,b). ‘Old Blush’ (syn. Parsons’ Pink China) was brought to Europe and North America in the eighteenth century from China and is one of the most influential genotypes in the history of rose breeding. Among other things, it introduced recurrent flowering into Western germplasm, which is an essential trait for the development of modern rose cultivars^15^. We validated our pseudo-chromosome scale genome assembly of ‘Old Blush’ using high-density genetic maps of multiple F1 progenies and synteny with *Fragaria vesca*. We delineated hallmark chromosomal features, such as the pericentromeric regions, through annotation of transposable element families and positioning of centromeric repeats using fluorescent in situ hybridization (FISH). This reference genome also allowed us to analyse the genetic diversity within the *Rosa* genus following a resequencing of eight wild species. Using genetic (F1 progeny and diversity panel) and genomic approaches, we were able to identify key potential genetic regulators of important ornamental traits, including continuous flowering, flower development, prickle density and self-incompatibility.

## Results

### Development of a high-quality reference genome sequence

We developed a haploid callus cell line (HapOB) using an anther culture at the mid-to-late uninucleate microspore developmental stage from the diploid heterozygous ‘Old Blush’ variety (Fig. 1c–e). The homozygosity of the HapOB line was verified with ten microsatellite markers distributed over the seven linkage groups (Supplementary Table 1). Flow cytometric analysis showed the HapOB callus to be diploid, suggesting that spontaneous genome doubling occurred during in vitro propagation.

A combination of Illumina short-read sequencing and PacBio long-read sequencing technologies was used to assemble the doubled haploid HapOB genome sequence. PacBio sequencing data (Supplementary Table 2) was assembled with CANU^16^, yielding 551 contigs (N50 of 3.4 Mb), representing a total length of 512 Mb. Of the obtained sequence, 95% is contained in only 196 contigs. The PacBio-based assembly was error corrected with Illumina paired-end reads: 37,300 single-nucleotide polymorphisms (SNPs) and 307,700 insertions and deletions (indels) were corrected, representing 341.1 kb (Supplementary Table 2). K-mer spectrum analysis (K = 25) suggested a genome size of 532.7 Mb (251.1 Mb of a unique genome sequence and 279.6 Mb of repetitive sequences), whereas flow cytometric analysis estimated a genome size of 1C = 568 ± 9 Mb. Thus, the assembled sequence represents 96.1% or 90.1%, respectively, of the estimated genome size. No major contamination was detected by screening for the predicted prokaryotic genes (Supplementary Table 3). Furthermore, only four contigs had low Illumina read mapping frequency, all of which were found to most likely encode plant proteins.

High-density female and male genetic maps were developed from a cross between *R.* *chinensis* ‘Old Blush’ and a hybrid of *Rosa wichurana* (OW). F1 progeny from this cross (*n* = 151) were genotyped with the WagRhSNP 68K Axiom array^17^ (Table 1 and Supplementary Table 4). Thirteen contigs, for which marker order clearly indicated assembly artefacts, were split before anchoring all 564 resulting contigs to the female and male genetic maps using a total of 6,746 SNP markers (Table 1). Of these, 196 contigs were anchored manually onto the seven linkage groups, mostly on both the female and the male genetic maps (174 and 143 contigs, respectively). In total, 466 Mb were therefore anchored onto the genetic maps and assembled into 7 pseudo-chromosomes representing 90% of the assembled contig length (Table 1 and Supplementary Fig. 1a). The remaining 368 contigs (52 Mb) were assigned to chromosome 0 (Chr0). The quality of the assembly of the seven pseudo-chromosomes was assessed using two independent genetic maps: the previously published integrated high-density genetic map (K5) based on 172 tetraploid F1 progeny^10^, and a newly developed high-density map based on 174 diploid F1 progeny from a cross between cultivar ‘Yesterday’ and *R.* *wichurana* (YW; see Supplementary Fig. 1b). The co-linearity between the pseudo-chromosomes and both linkage maps is excellent (Supplementary Fig. 2). In addition, the anchoring of the 386 contigs (52 Mb), currently assigned to Chr0, onto the K5 map and the YW map revealed that 39 contigs (total: 28.4 Mb) and 27 contigs (total: 24.1 Mb), respectively, can potentially be positioned onto the 7 linkage groups (Supplementary Fig. 2). However, because these genetic maps were created using independent genotypes that are not related to *R.* *chinensis* ‘Old Blush’, we chose a conservative approach by not incorporating these contigs into the pseudo-chromosome sequence of HapOB.

**Table 1:** Metrics of the alignment of the male and female genetic maps with the HapOB genome assembly

Linkage group	Genetic maps (no. of markers)		Chr.	No. of anchored markers used for anchoring		No. of anchored contigs					Pseudo-molecules
	Female (OB)	Male (W)		Female	Male	Female	Male	Manual integration	Cut	Excluded	Size (in bp)
1	715	195	1	587	146	18	14	18	1	1	64,770,848
2	1,114	303	2	1,001	249	14	18	20	–	–	75,129,302
3	528	564	3	477	498	20	25	31	–	1	46,843,630
4	227	404	4	191	334	12	18	20	–	–	59,004,735
5	1,031	362	5	866	275	40	29	37	2	1	85,885,663
6	1,153	254	6	1,010	186	43	20	43	–	1	67,395,200
7	863	241	7	743	183	27	19	27	–	–	67,081,725
–	–	–	Total without Chr0			174	143	196	–	–	466,111,103
–	–	–	0	–	–	387	418	368	–	–	52,404,850
Total:	5,631	2,323	–	4,875	1,871	561	561	564	–	–	518,515,953

The genetic maps were developed from a cross between ‘Old Blush’ (OB; female) and a hybrid of *R.* *wichurana* (W; male) using an Affymetrix SNP array. The initial size of the genome was 512 Mb and reached a final size of 518.5 Mb owing to the addition of 10,000 N between each contig to create the pseudo-molecules. N, any nucleotide.

### Positioning centromeres within the genome assembly

**Fig. 2:**
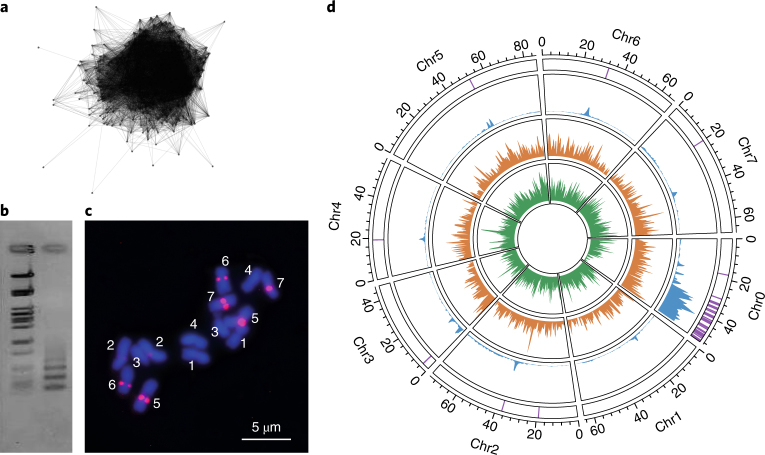
Identification of centromeric regions in the HapOB reference genome. **a**, The cluster CL226 identified by RepeatExplorer. **b**, Agarose gel electrophoresis of tandem repeat fragments amplified from the genomic DNA of HapOB using OBC226 PCR primers (right lane) along with the lambda-*Pst*I size ladder (left lane). Similar results were obtained in two independent experiments. **c**, FISH with carboxy tetramethylrhodamine (TAMRA)-labelled OBC226 oligo probes on *R.* *chinensis* metaphase chromosomes. Chromosome numbers are labelled from 1 to 7. Similar results were observed in at least 10 metaphase cells in two independent experiments. **d**, Circos representation of the distribution of OBC226 (purple), the pericentromeric region (blue), Ty3/*Gypsy* (orange) and Ty1/*Copia* repeat elements (green) along the seven pseudo-chromosomes and Chr0 (scale in Mb).

The centromeric regions were identified using both bioinformatic and cytogenetic methods. We discovered a highly abundant tandem repeat (0.06% of the genome with more than 2,000 copies per haploid genome) of monomers (159 bp long) that we call OBC226 (‘Old Blush’ centromeric repeat from RepeatExplorer cluster 226; Fig. 2a). PCR confirmed the tandem organization of this repeat (Fig. 2b). FISH analysis unambiguously confirmed the location of the repeat in the centromeric regions of four of the seven chromosomes: Chr2, Chr5, Chr6 and Chr7 (Fig. 2c). Mapping of the OBC226 repeat sequence revealed regions with high coverage on all HapOB pseudo-chromosomes except Chr1, which explains why no clear centromeric region could be detected on this chromosome (Fig. 2d). On Chr3 and Chr4, the copy number of OBC226 was probably too low to be detected by FISH. Furthermore, the core OBC226 centromeric repeats were flanked by other repetitive sequences, and these were unequally distributed along the chromosomes, with a clearly higher density in the core centromeric regions (Fig. 2d). These centromeric regions were also enriched in Ty3/*Gypsy* transposable elements. Taken together, these results confirm the position of the centromeric regions on the seven pseudo-chromosomes and reveal the high repeat sequence content, and low gene content, of the scaffolds currently assigned to Chr0.

### Annotation of the sequence

#### Coding genes

Based on the mapping of 723,268 transcript sequences (expressed sequence tag/complementary DNA and RNA sequencing (RNA-seq) contigs with a minimum size of 150 bp) onto the HapOB genome assembly, we predicted a total of 44,481 genes covering 21% of the genomic sequence length using Eugene combiner^18^. These include 39,669 protein-coding genes and 4,812 non-coding genes. Evidence of transcription was found for 87.8% of all predicted genes. At least one InterPro domain signature was detected in 86.5% of the protein-coding genes using InterProScan^19^, with 68.0% of the genes assigned to 4,051 PFAM gene families^20^. The quality of the structural annotation was assessed using the BUSCO v2 method based on a benchmark of 1,440 conserved plant genes^21^, of which 92.5% had complete gene coverage (including 5.3% duplicated ones), 4.1% were fragmented and only 3.4% were missing. This result can be compared to the analysis of the whole-genome assembly, which identified 95% complete genes and 3.6% missing genes. The set of predicted non-coding genes included 186 ribosomal RNA, 751 transfer RNA, 384 small nucleolar RNA, 99 microRNA, 170 small nuclear RNA and 3,222 unclassified genes (annotated as non-coding RNA) with evidence of transcription but no consistent coding sequence.

The number of predicted proteins in *Rosa* (39,669) is higher than the number of predicted proteins in *F.* *vesca* (28,588 predicted proteins^22^). By BLAST analysis, we identified 6,543 proteins that are rose specific. Among them, 5,867 proteins have no homologue in *Arabidopsis thaliana*. For these proteins, no functional information is available from closely related species and experimental evidence will be required to explore their role in roses. We also looked at whether the difference in the predicted number of proteins was owing to protein family expansion. Such a scenario was detected for some protein families, including nucleotide-binding site leucine-rich repeat (NBS-LRR) and cytochrome P450 (Supplementary Fig. 3).

#### Transposable elements

The REPET package^23^ was used to generate a genome-wide annotation of repetitive sequences of the HapOB genome (see Methods for details). Retrotransposons, also called class I elements, represent the largest transposable element genomic fraction (35.1% of the sequenced genome), of which long terminal repeat (LTR) retrotransposons represent 28.3%. *Gypsy* elements are more frequent than *Copia* (Supplementary Table 5a). Non-LTR retrotransposons long intersperced nuclear elements (LINEs) and potential short intersperced nuclear elements (SINEs) represent 5.0% of the sequence genome and class II elements (DNA transposons and Helitrons) represent 11.7% (Supplementary Table 5a). The remaining 15% include unclassified repeats (7.3%), chimaeric consensus sequences (1.9%) and potential repeated host genes (5.8%). We also identified Caulimoviridae copies representing 1.25% of the genome. Interestingly, one particularly abundant *Gypsy* Tat-like family was found in the genome assembly. The total copy coverage represents 3.4% of the genome. Tat-like elements are known to have an open reading frame (ORF) after the polymerase domains, and surprisingly in this case, the ORF corresponds to a class II transposase domain.

In a preliminary comparison between the transposable element annotation in HapOB and the *F.* *vesca* v2.0.a1 genome assembly (without manual curation) (Supplementary Table 5b), we found that retrotransposon elements represent the largest transposable element genomic fraction in *F.* *vesca* (13.91%), similar to rose. We found approximately twofold more copies for all transposable element families except SINE and unclassified in *Rosa* than in *Fragaria*. This indicates that the difference in genome size between *Rosa* and *Fragaria* is largely due to an expansion of the transposable element fraction.

### Synteny between *Rosa* and *F.* *vesca*

*Rosa* and *Fragaria* both belong to the Rosoideae subfamily of the Rosaceae^24^, having diverged around 50 million years ago^25^. Previous genetic studies have demonstrated that large macrosyntenic blocks are conserved between *Rosa* and *Fragaria*^10,26^. We compared the HapOB genome to the recently updated *F.* *vesca* genome^22^ to analyse the synteny in detail (Supplementary Fig. 4a). *R.* *chinensis* Chrs 1, 4, 5, 6 and 7 display strong synteny with *F.* *vesca* Chrs 7, 4, 3, 2 and 5, respectively. Consistent with previous suggestions^10^, a reciprocal translocation was detected between *R.* *chinensis* Chr 2 and 3 and *F.* *vesca* Chrs 6 and 1, respectively. Our results clarify the highly conserved synteny between *F.* *vesca* and *Rosa*, revealing only two major translocation events.

Within the Rosaceae family, the synteny is also well conserved between *Prunus* and *Rosa* (Supplementary Fig. 4c): *Rosa* Chr1 corresponds to *Prunus* Chr2, *Rosa* Chr4 corresponds to the end of *Prunus* Chr1, whereas *Prunus* Chrs 3, 5 and 8 correspond to large parts of *Rosa* Chrs 2, 6 and 7 respectively. Owing to the allopolyploid origin of *Malus*, the overall synteny is less clear, even if large blocks of synteny can be detected (Supplementary Fig. 4b).

### Genetic diversity with the genus *Rosa*

**Fig. 3:**
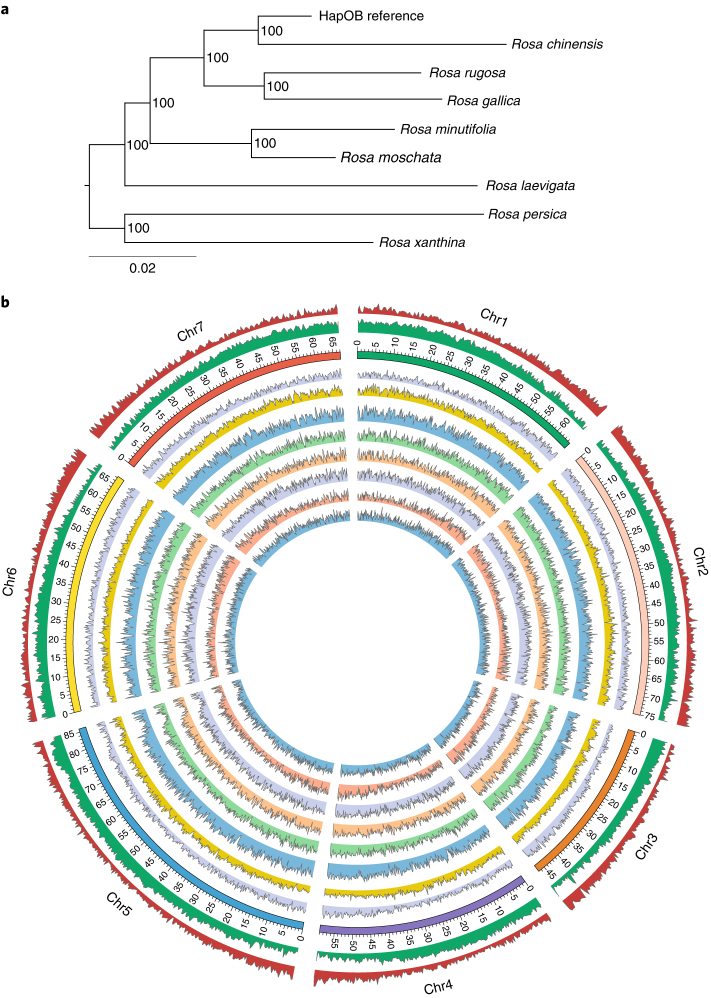
Resequencing of eight *Rosa* species. **a**, The phylogenetic relationships of the eight sequenced *Rosa* species and the reference genome HapOB, using a genome-wide set of homozygous SNPs. **b**, Analysis of genetic diversity in eight species of the *Rosa* genus along the seven pseudo-chromosomes of the HapOB reference sequence. Circles from outside to inside show: gene density (red), transposable element density (green), SNP density for *R.* *xantina* (purple), *R.* *chinensis* var. *spontanea* (yellow), *R.* *gallica* (blue), *R.* *laevigata* (light green), *R.* *moschata* (light orange), *R.* *rugosa* (light purple), *R.* *persica* (light red) and *R.* *minutifolia alba* (light blue). Scales are in Mb.

The 150 or more existing rose species belong to four subgenera. Excluding the subgenus *Rosa*, all subgenera contain only one or two species. We resequenced eight *Rosa* species (Table 2), representing three of the four subgenera (*Hulthemia*: *R.* *persica*, *Herperhodos*: *R.* *minutifolia* and *Rosa*). Within *Rosa*, we covered all of the main sections according to the latest phylogenetic analyses^27,28^ (Table 2) in the form of *R.* *chinensis* var. *spontanea*, *R.* *rugosa*, *R.* *laevigata, R.* *moschata, R.* *xanthina spontanea* and *R.* *gallica*. All are diploid species except *R.* *gallica*, which is tetraploid (Table 2). SNPs and indels were identified relative to the HapOB reference sequence (Fig. 3).

**Table 2:** Summary of resequencing and sequence variations (SNP and small indels) identified in eight *Rosa* species

*Rosa* species sequenced	Genome size (in Mb)	Classification		Ploidy	Flower colour	Flower morphology	Blooming seasonality	Geographical origin	No. of reads (millions)	No. of reads mapped (millions)	HapOB genome covered by the mapping (%)	Depth of coverage (in ×)^a^	No. of SNPs	SNP/density (no. per kb)	No. of small indels	Small indel density (no. per kb)
		Subgenus	Section													
*R.* *chinensis* var. *spontanea*	562	*Rosa*	*Chinenses*	2	Pink	Single	Once blooming	China	110	104	90	28	5,564,345	9.9	876,648	1.6
*R.* *gallica*	538	*Rosa*	*Gallicanae*	4	Pink	Single	Once blooming	Europe	231	218	90	73	11,280,831	21.0	2,430,138	4.5
*R.* *laevigata*	562	*Rosa*	*Laevigatae*	2	White	Single	Occasionally	China–Taiwan	100	92	70	31	6,327,292	11.3	1,195,164	2.1
*R.* *moschata*	554	*Rosa*	*Synstylae*	2	White	Single	Recurrent blooming	Asia Minor	92	86	71	29	5,862,043	10.6	1,417,766	2.6
*R.* *munitifolia alba*	416	*Hesperhodos*		2	White	Single	Once blooming	North America	96	89	69	30	5,270,249	12.7	1,208,933	2.9
*R.* *persica*	416	*Hulthemia*		2	Yellow	Single	Once blooming	Central Asia	114	100	56	34	5,602,086	13.5	1,218,337	2.9
*R.* *rugosa*	522	*Rosa*	*Cinnamomeae*	2	Pink	Single		Northern China–Japan–Korea	125	116	84	39	8,270,874	15.8	1,703,127	3.3
*R.* *xanthina spontanea*	391	*Rosa*	*Pimpinellifoliae*	2	Yellow	Single	Once blooming	Asia	95	85	60	28	5,642,595	14.4	1,316,384	3.4

^a^The depth of coverage is the ratio between the number of mapping base pairs (the number of mapping reads × read size) and the genome size.

The nuclear SNP-based phylogenetic tree of the eight species (Fig. 3a) is consistent with previous molecular analyses^27,28^. The clade, including *R.* *chinensis*, *R.* *gallica*, *R.* *moschata* and *R.* *laevigata*, fits with the *Synstylae* and allies clade previously found in a chloroplastic analysis^28^. The same is the case for *R.* *persica* and *R.* *xanthina*, both belonging to the *Cinnamomeae* and allies clade. However, *R.* *rugosa* and *R.* *minutifolia* show an uncertain position. In particular, *R.* *minutifolia*, which belongs to the *Hesperhodos* subgenus^27,28^, was expected to be closer to *R.* *persica* and *R.* *xanthina*. One of the possible explanations is that the resequenced *R.* *minutifolia* individual is actually the product of an interspecific cross, as it shows unexpected morphological characters, such as few prickles on the young flowering shoots and flowers clustering in inflorescences. Methodologically, the use of only homozygous SNPs may have caused bias, especially in *R.* *rugosa*, as most of its SNPs were in the heterozygous state (Supplementary Table 6).

The lowest SNP and indel density was found in *R.* *chinensis* var. *spontanea* (9.9 and 1.6 per kb, respectively). ‘Old Blush’ is described as an interspecific cross between *R.* *chinensis* var. *spontanea* and *R*. x *odorata* var. *gigantea*^6^, which is consistent with the relatively low sequence divergence of *R.* *chinensis* var. *spontanea* compared to the HapOB reference sequence. The highest SNP and indel density was found in *R.* *gallica* (21.0 and 4.5 per kb, respectively); this could be the result of the (allo)tetraploidy of this species^29^, as shown by its high proportion of heterozygous SNPs (74%; Supplementary Table 6).

As expected, the majority (79.2–89.0%) of the SNPs were located in non-coding regions (Supplementary Table 6). Only 3–7% of the SNPs were located in exons, of which half were synonymous, in line with other species (for example, tomato^30^). The different species displayed varying levels of homozygosity (homozygous SNPs ranging from 79.2% in *R.* *persica* to 26.0% in tetraploid *R.* *gallica*; Supplementary Table 6). The number of small indels was higher (between 876,648 and 2,430,123) than *Malus*, with an average of 346,498 indels^31^, suggesting a higher level of diversity within the *Rosa* genus.

### Analysis of the genetic control of important traits

**Fig. 4:**
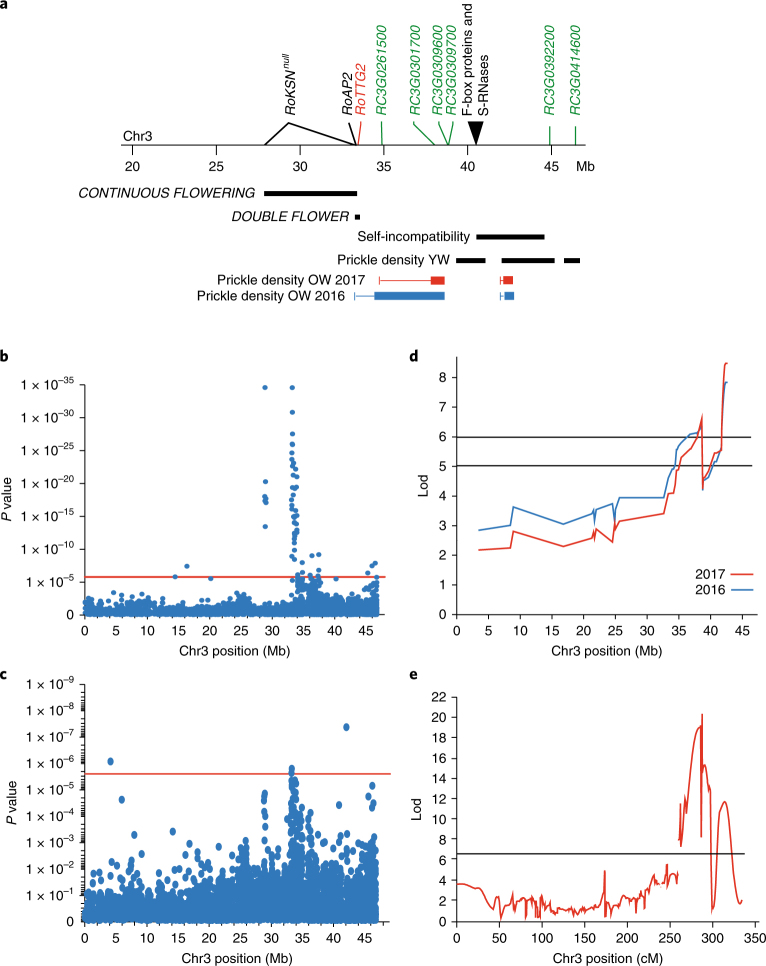
A region at the end of Chr3 controls important ornamental traits. **a**, Major genes and QTLs that control continuous flowering, double flower, self-incompatibility and prickle density are shown together with candidate genes for each trait. Detailed analyses per locus are described in Supplementary Figs. 5, 7, 9 and 10, respectively. For prickle density in OW progeny (OW2017 and OW2016), the boxes represent the 1-LOD (log of the odds ratio) interval and the lines the 2-LOD interval. **b**,**c**, GWAS analysis showing the *P* values of the association between SNPs positioned along Chr3 and the number of petals, indicating regions that control the number of petals. The petal number is considered as a qualitative trait (simple versus double flowers; GLM) (**b**) or as a quantitative trait (MLM) (**c**). The horizontal red line shows Bonferroni-corrected significance levels (1.78 × 10^–6^). Other significant associations detected by GWAS are shown in Supplementary Fig. 12. *n* = 96 cultivars with 3 flowers scored by cultivar. **d,e**, QTL analysis for prickle density in two F1 progenies using the OW mapping population based on scoring from 2016 and 2017, n = 151 individuals (**d**), and the YW mapping population, *n* = 174 individuals (**e**). Lod, log likelihood ratio.

This new reference sequence is an important tool to help decipher the genetic basis of ornamental traits, such as blooming (including continuous flowering, flower development and the number of petals), prickle density on the stem and self-incompatibility. We studied the genetic determinism in (1) two F1 progenies (151 individuals obtained from the OW progeny and 174 individuals obtained from the YW progeny), and (2) a panel of 96 rose cultivars originating from the nineteenth to the twenty-first century^32,33^. Our data demonstrate that important loci controlling continuous flowering, double flower morphology, self-incompatibility and prickle density were predominantly localized in a single genomic region of Chr3 (Fig. 4a).

#### Detection of a new allele controlling continuous flowering in rose

Most species of roses are 'once flowering'. In rose, continuous flowering is controlled by a homologue of the *TERMINAL FLOWER 1* (*TFL1*) family, *RoKSN*, located on Chr3 (ref. ^34^). The continuous flowering phenotype is due to the insertion of a *Copia* retrotransposon element in the *RoKSN* gene. The continuous flowering rose ‘Old Blush’ was previously proposed to be *RoKSN*^*Copia*^*/RoKSN*^*Copia*^ at the *RoKSN* locus^34^. This *Copia* element corresponds to the RLC_denovoHm-B-G10244-Map11 retrotransposon. We identified 34 insertions of this transposable element in the HapOB genome, of which 11 are full length (Supplementary Table 7a). The element is inserted into three genes, all of which are disrupted. The 3′ and 5′ LTRs of the full-length elements are >99% similar (Supplementary Table 7a), suggesting a recent insertion, as previously proposed for the element inserted in *RoKSN*^34^.

Here, quantitative trait locus (QTL) analysis in the OW progeny identified the *CONTINUOUS FLOWERING* locus on Chr3 (Fig. 4a), as expected, but we were unable to detect the *RoKSN* gene in the annotated HapOB genome. Detailed analysis of *RoKSN* allele segregation in the OW progeny revealed the existence of a null allele, in which *RoKSN* is deleted (see Supplementary Table 8 for further details). The diploid ‘Old Blush’ parent of the OB mapping population is therefore hemizygous *RoKSN*^*Copia*^*/RoKSN*^*null*^, and the *RoKSN*^*null*^ allele is present in the HapOB genome sequence.

Interesting parallels exist between rose and *F.* *vesca* because *F.* *vesca* also exhibits both the once flowering and the continuous flowering phenotypes. In strawberry, a 2-bp deletion in the *TFL1* homologue causes a shift from once flowering to continuous flowering^34,35^. Synteny analysis revealed four orthologous syntenic blocks in the *RoKSN* gene region, here called blocks A–D (Supplementary Fig. 5). We detected a pattern of conserved gene content in combination with genome rearrangements between different *Rosa* species and the published genome sequence of *F.* *vesca*^36^ where the synteny with *F.* *vesca* is broken at the *FvKSN* location. The *FvKSN* gene is located between the A and B blocks in *F.* *vesca*. The A block is inverted in the HapOB genome, and the C and D blocks are inserted between the A and B blocks. In *R.* *multiflora*^14^ and *R.* *laevigata* (see Methods for the partial *R.* *laevigata* genome sequence assembly), which are both once flowering, the *RoKSN*^*WT*^ allele is present and synteny is conserved with *F.* *vesca* (Supplementary Fig. 5). Taken together, these data indicate that the *RoKSN*^*null*^ allele is the result of a large rearrangement at the *CONTINUOUS FLOWERING* locus, leading to the complete deletion of the *RoKSN* gene. The *RoKSN*^*null*^ allele represents a novel allele responsible for continuous flowering, which has not been previously described.

#### Double flower

The number of petals is an important ornamental trait, and roses with higher numbers of petal (‘double flower’) have traditionally been selected. Through a study of mutant lines (sports), the change in petal number was attributed to a homeotic conversion in organ identity, with stamens converted into petals^37^. The genetic basis of the double flower trait is complex, with a dominant gene (*DOUBLE FLOWER*) controlling simple versus double flower phenotypes and two QTLs controlling the number of petals on double flowers^38^. Here, we combined the genome sequence with segregation data of four different F1 progenies to confine the putative location of the *DOUBLE FLOWER* locus (Supplementary Table 9) to a 293-kb region of Chr3 (between position 33.24 Mb and 33.53 Mb; Fig. 4a). Using a genome-wide association study (GWAS) approach with a panel of 96 cultivated roses, we detected a strong association with simple versus double flowers in the same region (between position 33.08 Mb and 33.94 Mb; Fig. 4b). A second significant peak was located at a distance of 5 Mb, which may correspond to a secondary locus influencing this trait.

The 293-kb region contains 41 annotated genes. Among these, half are expressed during the early stages of floral development (Supplementary Table 10). By excluding genes expressed in later floral stages (with completely open flowers), we retained four candidate genes: an F-box protein (RC3G0245100), a homologue of *APETALA2/TOE* (RC3G0243000), a Ypt/Rab-GAP domain of the gyp1p superfamily protein (RC3G0245000) and a tetratricopeptide repeat-like superfamily protein (RC3G0243500) (Supplementary Table 10).

Concerning double flowering, ‘Old Blush’ is heterozygous for the *DOUBLE FLOWER* locus. Sequencing both alleles of the four selected candidate genes in ‘Old Blush’ revealed only minor modifications for RC3G0245100, RC3G0245000 and RC3G0243500 (Supplementary Fig. 6a–c, respectively). However, concerning the *APETALA2/TOE* gene (RC3G0243000), we detected a 1,426-bp insertion in intron eight (Supplementary Fig. 7a). The insertion showed high similarity to an unclassified transposable element (annotation noCAT_denovoHM-B-R7962-Map20; Supplementary Table 5c). This repeat element is present 62 times in the HapOB genome, of which 20 insertions are full length and 4 are located in gene introns (Supplementary Table 7b). Apart from this insertion and a few SNPs, no other differences were detected between the two alleles. In the OW progeny, all individuals that carry the transposable element insertion allele display the double flower phenotype (see Supplementary Table 11 for further details).

Phylogenetic analysis showed that RC3G0243000 belongs to the *APETALA2/TOE* clade within the *AP2/ERF* subfamily^39^ (Supplementary Fig. 7d). Like all members of the AP2 clades, the protein encoded by RC3G0243000 contains two conserved AP2 domains and a conserved putative *miRNA172* binding site (Supplementary Fig. 7b,c). The genomic position, expression analysis, protein sequence data and predicted deleterious effect of the insertion in intron 8 suggest that the *APETALA2/TOE* gene is a good candidate for the *DOUBLE FLOWER* locus. *APETALA2/TOE* has a central role in the establishment of the floral meristem and in the specification of floral organs^40–43^. *APETALA2* was classified as a class A floral homeotic gene, which specifies sepal identity if expressed alone and petal identity if expressed together with class B genes^44^. Furthermore, *AP2/TOE3* repressed *AGAMOUS* expression (a class C gene) in the two outer floral whorls in the floral meristem^42^ (reviewed in ref. ^45^). In rose, a reduction in the levels of *RhAGAMOUS* transcripts was proposed to be the basis of the double flower phenotype^37^. We hypothesise that misregulation of the rose *APETALA2/TOE* homologue (due to the presence of the transposable element) is responsible for the *RhAGAMOUS* transcript level reduction, leading to the double flower phenotype.

Interestingly, a GWAS approach for petal number (a quantitative analysis) in a panel of tetraploid and double flower varieties^33^ revealed that the most significant QTL is also located at the *DOUBLE FLOWER* locus (Fig. 4c). Several markers in this cluster display significant dose-dependent effects on the number of petals. One of these markers, RhK5_4359_382 (at position 33.55 Mb), was analysed via the Kompetitive allele specific PCR (KASP) technology both in the original association panel of 96 cultivars and in an independent panel of 238 tetraploid varieties and showed the same effect in both populations (Supplementary Fig. 8a,b). Two other markers (RhK5_14942 and RhMCRND_760_1045) were also tested on the 96 cultivars by KASP technology and revealed the same pattern (Supplementary Fig. 8c,d). This demonstrates a dual role of the *DOUBLE FLOWER* locus in rose: it controls both the double flower phenotype (double versus single flowers) and the number of petals. Given that the petal number QTL was detected in several panels of unrelated rose genotypes, it seems that this locus acts independently of the genetic background.

#### Self-incompatibility

As described for other Rosaceae species^46–48^, in some diploid roses, self-incompatibility is caused by a gametophytic SI (self-incompatibility) locus. This locus is most likely composed of genes encoding S-RNases and F-box proteins, which represent the female- and male-specific components, respectively. Previous approaches have failed to characterize the *Rosa* Sl-locus genes owing to the low sequence similarity between S-RNase genes across species and the existence of multiple genes for both S-RNases and F-box proteins. A screen for S-RNase and F-box homologues in the HapOB genome sequence identified a region of 100 kb on Chr3 that contains three genes coding for S-RNAses and four genes for S-locus F-box proteins (Fig. 4a and Supplementary Fig. 9a). This region is syntenic with the SI locus in *Prunus persica* (Supplementary Fig. 9b). One of the S-RNases (*S*-*RNase36*) was expressed in pistils of ‘Old-Blush’ flowers. Of the F-box genes, *Fbox38* accumulated in the stamens (Supplementary Fig. 9c,d). Hence, this region fulfils the requirements of a functional S-locus.

This region is consistent with previous data on segregation of the self-incompatibility phenotype in a diploid rose population, in which the self-incompatibility phenotype was analysed by generating a bi-parental progeny and backcrossing individual progeny to both parents^49^. We generated a marker for an orthologue of the S-RNase gene (*SRNase30*) expressed in pistils of ‘Old Blush’ that co-segregates with the S-locus at a distance of 4.2 cM. The large number of recombinants might be explained by incomplete expression of self-incompatibility (leaky phenotypes) in some individuals of the progeny, a phenomenon that is also observed in, for example, *Solanum* populations^50^.

#### Prickle density

We investigated the genetic regulation of prickle density in rose. In two F1 progenies, QTLs were detected on Chr3. In the OW and YW progenies, a large region of significant association was detected between position 31.2 Mb and the end of the chromosome on both male and female maps (Fig. 4d,e, respectively). In both populations, two peaks were clearly detected, which probably correspond to two neighbouring QTLs (Fig. 4a,d,e). Through a GWAS approach, we detected a strong association between SNPs and the presence of prickles between positions 31.0 Mb and 32.4 Mb (Supplementary Fig. 10a). In rose, prickles originate as a deformation of glandular trichomes in combination with cells from the cortex^51^. We have looked for homologues of candidate genes controlling trichome initiation and development identified in *A.* *thaliana*^52^. Screening the QTL region on Chr3 of HapOW for gene family members of these candidate genes revealed several WRKY transcription factors, of which RC3G0244800 (positioned at 33.40 Mb; Fig. 4a) shows strong similarity with *AtTTG2* (*TESTA TRANSPARENT GLABRA2*), which is involved in trichome development in *Arabidopsis*^53^ (Supplementary Fig. 10b). We studied the expression of the rose *TTG2* homologue (*RcTTG2*) in three different individuals of the OW progeny with different prickle densities (absence, medium- and high-density prickles on the stem; Supplementary Fig. 10c). The *RcTTG2* transcript accumulated at higher levels in stems presenting prickles, suggesting that *RcTTG2* is a positive regulator of prickle presence in rose. This *TGG2* homologue represents a good candidate for the control of prickles in rose.

## Discussion

We have produced a high-quality reference rose genome sequence that will represent an essential resource for the rose community but also for rose breeders. Using this new reference sequence, we have analysed important structural features of the genome, including the position of the centromeres (Fig. 2) and SNP and indel frequencies (Fig. 3).

Taking advantage of this new high-quality reference sequence, rose is set to become a model species to study ornamental traits. For example, rose was previously used to study scent emission, leading to the discovery of a new pathway for the synthesis of monoterpenes^54^. Here, using a combination of genomic and genetic approaches (F1 progenies and GWAS diversity panel), we have demonstrated that this new reference sequence can be used to analyse loci controlling ornamental traits, such as continuous flowering, double flower, self-incompatibility and prickle density (Fig. 4). We have identified and characterized candidate genes for these traits. We propose that a rose *APETALA2/TOE* homologue controls the switch from simple to double flower and, unexpectedly, also the number of petals within double flowers. Further analyses are necessary to validate the function of these genes. The analyses were done in diploid roses but also in tetraploid roses, allowing direct implementation in rose breeding materials, with the development of diagnostic markers as we demonstrated for petal number. For this economically crucial trait, we have developed a genetic marker that permits the prediction of petal number, which we validated on a large panel (Supplementary Fig. 8). This represents a good example of how the development and release of the rose genome sequence can accelerate gains in rose breeding.

Cultivated roses have an allopolyploid background but segregate mainly tetrasomically^10,55^. Hence, rose is a unique model for polyploidization and chromosome pairing mechanisms, which can now also be investigated at the molecular level. This reference sequence opens the way to genomic and epigenomic approaches to study important traits, providing an essential bridge between this and other plant species.

## Methods

### Development of haploid ‘Old Blush’ callus

Young flower buds of ‘Old Blush’ (Fig. 1c) with microspores at a mid-to-late uninucleate developmental stage (Fig.  1d) were collected in a greenhouse, wrapped in aluminium foil and stored in the dark at 4 °C for 25 days. These were then surface sterilized in 70% ethanol for 30 s and in sodium hypochlorite solution (2.9° active chloride) for 15 min followed by rinsing three times in double-distilled sterilized water.

Anthers were aseptically removed using binoculars and ground in starvation B medium^56^ with minor modifications (pH 6 and 0.1 M sorbitol) for 2 min using a MSE homogenizer (Measuring & Scientific Equipment) set at 10,000 r.p.m. Anthers were then collected on 50-µm mesh filters, covered with a fine layer of fresh modified starvation B medium and incubated for 24 h at 22 °C in darkness. Anthers were transferred on MS medium containing 30 g l^−1^ sucrose, 0.5 mg l^−1^ BAP (6-benzylaminopurine) and 0.1 mg l^−1^ NAA (naphthaleneacetic acid) in 12-well culture plates. Plates were incubated in darkness at 23 °C/19 °C (16 h/8 h), taking care not to move the boxes or expose them to light for 80 days to induce somatic embryo formation. Somatic embryos were isolated from the anthers and transferred on the same medium in petri dishes with filter paper in 4-week intervals until the production of callus (Fig. 1e). Then, callus was multiplied on the same medium in the dark until enough material for DNA extraction was produced. Homozygosity was verified using ten previously described microsatellite markers^57^.

Genome sizes and ploidy levels were analysed on a flow cytometer, PASIII (488-nm, 20-mW laser; Partec). The Cystain absolute PI reagent kit (Sysmex) was used for sample preparation. *Solanum lycopersicum* ‘Stupické polni tyckove rane’ (1,916 Mb/2C) was used as an internal standard.

### Genome sequencing and assembly

#### DNA extraction for PacBio and Illumina sequencing

Callus tissues of the haploid ‘Old Blush’ HapOB line was kept in the dark for 3 days prior to DNA extraction to reduce chloroplast DNA contamination. DNA extraction was performed on 1 g HapOB callus tissue as described previously^58^. In total, approximately 30 mg genomic DNA was obtained in several batches for the preparation of three independent single-molecule real-time (SMRT) bell libraries. For the first library, genomic DNA was sheared by a Megaruptor (Diagenode) device with 30-kb settings. Sheared DNA was purified and concentrated with AMpureXP beads (Agencourt) and further used for SMRTbell preparation according to the manufacturer’s protocol (Pacific Biosciences; 20-kb template preparation using BluePippin (Sagesscience) size selection system with a 15-kb cut-off). Two additional libraries were made excluding the DNA shearing step, but with an additional initial damage repair. Size-selected and -isolated SMRTbell fractions were purified using AMPureXP beads and finally used for primer and polymerase (P6) binding according to the manufacturer’s binding calculator (Pacific Biosciences). Three library DNA–polymerase complexes were used for Magbead binding and loaded at 0.16, 0.25 and 0.20 nM on-plate concentrations, using 12, 7 and 8 SMRT cells, respectively. Final sequencing was done on a PacBio RS-II platform, with a 345- or 360-min movie time, 1 cell per well protocol and C4 sequencing chemistry. Raw sequence data were imported and further processed on a SMRT Analysis Server v2.3.0.

For Illumina sequencing, approximately 200 ng genomic DNA was sheared in a 55-µl volume using a Covaris E210 device to approximately 500–600 bp. One library with an insert size of 720 bp was made using Illumina TruSeq Nano DNA Library Preparation Kit according to the manufacturer’s guidelines. The final library was quantified by Qubit fluorescence spectrophotometry (Invitrogen) and the library fragment size range was assessed by Bioanalyzer High Sensitivity DNA assay (Agilent). The library was used for clustering as part of two lanes of a paired-end flow cell v4 using a Cbot device and subsequent 2 × 125 paired-end sequencing on a Hiseq2500 system (Illumina). De-multiplexing was carried out using Casava 1.8 software.

#### Genome assembly, polishing and contamination assessment

All sequence data generated that were derived from 27 SMRT cells containing 19.2 Gb of reads larger than 500 bp were assembled with CANU hierarchical assembler v1.4 (ref. ^16^) (version release r8046). In general, default settings were used except ‘corMinCoverage’, which was changed from 4 to 3, ‘minOverLapLength’, which was increased from 500 to 1,000, and ‘errorRate’, which was adjusted to 0.015. The assembly was completed on the Dutch National e-Infrastructure with the support of SURF Cooperative using 2,024 CPU hours (Intel Xeon Haswell 2.6 GHz) for the complete CANU process. Illumina paired-end (2 × 125 bp) reads were mapped onto the genome assembly using Burrow-Wheeler aligner maximum exact match (BWA-MEM)^59^. Pilon^60^ was then used to error correct the assembly. This procedure was repeated three times iteratively.

For contamination assessment, prokaryotic genes were predicted on the contigs using MetaGeneAnnotator^61^. The number of genes per nucleotide was computed for every contig. Furthermore, Illumina reads were mapped on the contigs using BWA-MEM^62^. The number of mapped reads per nucleotide was computed for every contig. Contigs with a low Illumina read mapping frequency were aligned against the GenBank non-redundant protein database using BLASTX.

### Development of high-density genetic maps and GWAS analysis

#### Plant material

A diploid F1 population of 151 individuals (OW) was obtained by crossing *R.* *chinensis* ‘Old Blush’ and a hybrid of *R.* *wichurana* obtained from Jardin de Bagatelle (Paris, France). This population was planted at the INRA Experimental Unit Horti (Beaucouzé, France).

A diploid F1 population of 174 individuals (YW) was obtained from a cross between ‘Yesterday’ and *R.* *wichurana* (the extended population as used in ref. ^63^). This population was planted at the ILVO (Melle, Belgium).

The tetraploid K5 cut rose mapping population consisted of 172 individuals obtained from a cross between P540 and P867. It was planted in Wageningen, the Netherlands, and was previously used in various QTL studies^64,65^.

The association panel comprised 96 cultivars, of which 87 were tetraploid, 8 were triploid and 1 was diploid, selected to reduce the genetic relatedness between genotypes^33^. Plants were cultivated in a randomized block design, with three blocks comprising one clone of each genotype both in the greenhouse and at an experimental field location at Leibniz Universität Hannover, Germany. For marker validation, an independent population of 238 tetraploid varieties was used that was cultivated in a field plot of the Federal Plant Variety Office in Hannover, Germany. Plants of the association panel and the phenotypic data are described in Supplementary Table 12.

#### Genetic map construction

The construction of the different genetic maps from F1 progenies (OW, YW and K5), the KASP assay for SNP validation and the development of a sequence characterized amplified region (SCAR) marker for the SI locus are described in Supplementary Methods.

#### GWAS analysis

The GWAS analyses for petal numbers and prickle density were performed in TASSEL 3.0 (ref. ^66^) as described previously^33^. Trait marker association for petal number was analysed using the mixed linear model (MLM) and 39,831 markers (petal as a quantitative trait with the Q + K model), including a fixed effect as the population structure matrix (Q) and random effect as the kinship matrix (K). Significance thresholds were corrected for multiple testing by the Bonferroni method using the number of contigs (19,083) as a correction factor, resulting in a significance threshold of 1.78 × 10^–6^. The kinship matrix used in the MLM was calculated for 10,000 SNP markers with the software SPAGeDi 1.5 (Zitat) as described previously^33^. For the GWAS analysis of prickles and petals with the general linear model (GLM) in TASSEL 3.0 (ref. ^66^), 63,000 markers were analysed. Petals and prickles were set as qualitative traits (1 and 0 to indicate presence or absence, respectively), and the analysis was performed without any correction for population (Q + K). Significance thresholds in the GLM were corrected by the number of contigs (28,054) to 1.78 × 10^–6^.

### Alignment of the HapOB rose genome with the OW genetic maps

The alignment of the genetic and physical maps was done in two steps. First, the HapOB sequence was aligned to the integrated genetic maps to detect problems of assembly (contigs that are present on two linkage groups). Second, to precisely order and orient the contigs on each linkage group, the alignment was done separately on the male and female maps and manually integrated.

During the first step, 7,822 out of a total of 7,840 SNP markers were positioned by mapping the corresponding 70-bp probes onto the HapOB genome sequence using Blat v.35 (ref.^67^). Markers with more than one best hit were eliminated. Out of the 7,360 remaining markers, 6,808 passed the mapping quality filter (≥95% match and ≤4% mismatch). Of these, 6,746 markers belonging to the most common linkage group on their respective contigs were conserved and described as 'concordant' markers. Only contigs with more than one of these markers were retained.

During the second step, the mapping and anchoring were done independently on the male and female maps (Table 1). The procedure and conditions were the same as for the first mapping. Only concordant markers were kept (4,875 (87%) and 1,871 (81%) for the female and male map, respectively). We positioned and oriented the different contigs manually (Supplementary Table 13). When a contig spanned several loci, its order and position were clear. However, for some contigs, genetic maps did not resolve the orientation problems. In these situations, we used the synteny between *Rosa* and *F.* *vesca*^10^. The strategy used to position and orient contigs is described in Supplementary Fig. 11. The position and orientation of the contigs are listed in Supplementary Table 13.

Concerning the K5 integrated genetic map, among the 25,695 SNP markers present, 20,706 SNPs (80.6%) could be positioned on the HapOB genome sequence by BLAST of the SNP-flanking marker sequences (Supplementary Fig. 2a).

### Centromere region identification and FISH

Three complementary tools were used to identify centromeric tandem repeats and to estimate their abundance in the *R.* *chinensis* ‘Old Blush’ genome: Tandem Repeat Finder (TRF^68^), TAREAN^69^ and RepeatExplorer^70^, each with default settings, and the output was parsed using custom python scripts. All tandem repeats identified by TRF were subjected to all-against-all BLAST to cluster similar repeats and to estimate abundance (the total number of tandem repeat cluster copies) in the genome. Paired reads were quality filtered and trimmed to 120 bp for analysis by RepeatExplorer (0.5 M read pairs) and TAREAN (1.3 M read pairs). RepeatExplorer cluster CL226 had the globular-like shape specific for tandem repeats. The corresponding monomer repeat sequence was identified by analysing the contigs of this cluster with TRF. The identical tandem repeat was also identified by TAREAN and TRF. To determine the location of the CL226 tandem repeat cluster in the genome assembly, 275 M paired-end genomic reads of ‘Old Blush’ were mapped onto the contigs from RepeatExplorer cluster CL226, using Bowtie2 (ref. ^71^) with parameter -k 1 to select read pairs with high similarity to the CL226 repeat. Selected read pairs were then split into two groups: reads that matched the CL226 repeat sequence itself and reads that matched the flanking genome sequence. Both groups of reads were separately mapped onto the genomic scaffolds using Bowtie with parameters -a 1 and -N 1. The distribution of the two sets of CL226 reads was visualized using the circlize package^72^ of R Bioconductor^73^. Mitotic chromosome slides were prepared with the 'SteamDrop' method^74^ using young root meristems of *R*. *chinensis* ‘Old Blush’. Two oligonucleotide probes (5′-TTGCGTTGTTCTAGTGACATTCA-TAMRA-3′; 5′-ACCCTAGAAGCGAGAAGTTTGG-TAMRA-3′) were used for FISH, as previously described^75^. DRAWID^76^ was used for chromosome and signal analysis.

### Annotation of the rose genome

Gene and transposable element annotations are described in Supplementary Methods.

### Diversity analysis

The plant material originated from ‘Loubert Nursery’ in Rosier-sur-Loire, France (*R.* *persica)*, from ‘Rose Loubert’ rose garden in Rosier-sur-Loire, France (*R.* *moschata*, *R.* *xanthina spontanea* and *R.* *gallica*) and from ‘Roseraie du Val de Marne’, Haÿ-Les-Roses, France (*R.* *chinensis* var. *spontanea*, *R.* *rugosa*, *R.* *laevigata* and *R.* *minutifolia alba*).

Illumina paired-end shotgun indexed libraries were prepared from 3 μg DNA per accession, using the TruSeq DNA PCR-Free LT Kit (Illumina). Briefly, indexed library preparation was performed with low-sample protocol with a special development to reach an insert size of 1–1.5 kb. DNA fragmentation was performed by AFA (Adaptive Focused Acoustics) technology on the focused ultrasonicator E210 (Covaris). All enzymatic steps and clean up were done according to the manufacturer’s instructions, apart from the fragmentation and sizing steps. Paired-end sequencing using 2 × 150 sequencing-by-synthesis cycles was performed on a HiSeq 2000/2500, Rapid TruSeq V2 chemistry (Illumina) running in rapid mode using on-board cluster generation (according to the manufacturer’s instructions). For some read sets, a low enrichment of libraries with five PCR amplification cycles was performed.

Cutadapt and FASTX toolkit software were used for quality control (Q > 30), and adapter trimming and high-quality reads were considered for further analysis. To identify the SNPs and indels in each species, filtered paired-end reads were mapped against the HapOB reference using BWA with default parameters^77^. The BWA software produced highly accurate alignment compared to other software. Unmapped and duplicated reads were removed using SAMtools and the Picard package, respectively^78^. Furthermore, reliable mapped reads were used for base quality score recalibration and indel realignment using the Genome Analysis Toolkit (GATK) software^79^. We then called variants individually on each sample using the HaplotypeCaller/GATK. The identified SNPs and indels were filtered out on the bases of a minimum read depth of 20 and SNP quality (Q) ≥ 40. The genomic distribution of SNPs and indels was analysed by calculating their frequency over each 200-kb interval on each HapOB chromosome. Circos was used to visualize the distribution of SNPs and indels on each HapOB chromosome. SnpEff and SnpSift^80,81^ were used to annotate the effects of SNPs and identify the potential functional effects of amino acid substitution on corresponding proteins, respectively.

To infer phylogenetic relationships between *Rosa* species, homozygous SNPs from each VCF file were merged using GATK CombineVariants and parsed to build a SNP alignment using VCFtools and our own scripts. A maximum likelihood analysis was performed using RAxML v8.1.5 with 100 bootstrap replicates^82^. As the SNP alignment contains only variable sites, an ascertainment bias correction was applied to the GTRGAMMA model of substitution^83^. The resulting phylogenetic tree was rooted on *R.* *persica*, which was purported to be the most divergent *Rosa* species^84^.

To conduct the synteny analysis between the HapOB reference sequence and *F.* *vesca*, orthologous genes were identified using reciprocal BLAST with an e-value of 1 × 10^5^ (ref. ^85^), v = 5 and b = 5. The protein sequences and annotation for *F.* *vesca* (v2.0.a1) were downloaded from the GDR database (https://www.rosaceae.org/). The output of the BLAST tool was used in the McSCANX tool to identify syntenic regions between the genomes^86^. The circos software^87^ was used to visualize the syntenic regions between two genomes. In addition, an analysis of microsynteny was performed between *R.* *chinensis* ‘Old blush’ and *F.* *vesca* for Chr3 to see the conserved region near the *RoKSN* locus using Symap software^85^.

Good-quality and pre-processed Illumina reads of *R.* *laevigata* were used for assembly. Genomic sequence reads were assembled using SPAdes (v3.11.1) with a k-mer value of 63 (ref. ^88^).

### Morphological traits

#### Petal number

For the OW and YW populations (151 and 174 individuals, respectively), the number of petals per flower was counted using 5 or up to 10 independent flowers, respectively. In roses, single flowers typically have five petals. Flowers with fewer than eight petals were considered as simple flowers, whereas those with eight or more petals were considered as ‘double’ flowers.

For the GWAS panel, the number of petals was counted for three flowers on each of the three clones from greenhouse-grown plants, and the arithmetic means were calculated for each genotype.

#### Prickle number

In the OW and YW populations, the length of a stem part with four internodes was measured in the middle of a stem (between the fifth and seventh internodes). Prickles were counted on four internodes. The prickle density was expressed as the number of prickles per internode. For each genotype, three stems were measured and counted.

For the GWAS panel, prickle density was calculated as the arithmetic mean of the number of prickles between the third and fourth node of newly developed shoots. For each genotype, three shoots were counted from three replicates in a randomized block design.

#### Expression analysis

For *TTG2* expression analysis, three individuals of the OW progeny were selected according to prickle density: OW9068 (no prickles), OW9155 (low density) and OW9106 (high density). The terminal part of young stems was harvested in spring 2016 from field-grown plants (two biological replicates). RNA extraction, cDNA synthesis, qPCR (three technical replicates) and relative quantifications were performed as previously described^89^. Calibration was done using *TCTP* and *UBC* genes. The following primers were used to amplify *TTG2* (RcTTG2-1-F: CCTCAAACCCAGGAGCATC and RcTTG2-1-R: CAACAGCTTGATCCCTGAGAG).

Organ-specific expression of candidate self-incompatibility genes were tested using RNA extracted from the stamens and pistils of three flower buds and five open flowers and the terminal leaflets of three young leaves, sampled from an individual of ‘Old Blush’ in August 2017. RNA extraction was carried out according to previously published protocols^37^. cDNA synthesis and RT–PCR were performed with the PrimeScript RT reagent Kit with genomic DNA Eraser and EmeraldAmp PCR Master Mix (TaKaRa) according to the manufacturer’s protocols. The following primers (5′ to 3′) were used to amplify seven candidate genes and a house-keeping gene: *SRNase26* (F1: TGCAGCCAACACATACGATT and R1: GCAAGAAGATCGGCGTAGTC), *SRNase30* (F1: TGTTCAACAATGGCCGATAA and R1: TGCACATAAGCGAAGGAGTG), *SRNase36* (F1: TGTGGTAACAGCTGCAAAGC and R1: TCAACCACGTTTTTGCCATA), *Fbox29* (F2: TGACTATTTTCTATTGCGCTTGAG and R1: CACCACAAAAAGGATAACAAGAC), *Fbox31* (F1: TTTGCTATGAAAATGATAACAACAG and R1: AACCCCATGGTTTCATTAAGTA), *Fbox38* (F1: GACTACTCTCCTTTGGCCTGAA and R1: CTACAGCTGCAGAATCATTTGAC), *Fbox40* (F1: CGTCCAATATCTCTACTCAATGGT and R1: CCTCTTCTTGGTGAGTCTGAAAT) and *RoTCTP* (F2: AAGAAGCAGTTTGTCACATGG and R2: TCTTAGCACTTGACCTCCTTCA).

### Reporting Summary

Further information on experimental design is available in the Nature Research Reporting Summary linked to this article.

### Code availability

The R code used for pairwise maximum likelihood recombination and lod score calculations is available through CRAN (https://CRAN.R-project.org/package=polymapR). The R code used to infer phylogenetic relationships is available on request from the corresponding author^90^. The python scripts used for centromeric region identification are available on request from the corresponding author.

### Data availability

All the genome data have been made available on a genome browser (https://iris.angers.inra.fr/obh/) and in the public GDR database (https://www.rosaceae.org/species/rosa/chinensis/genome_v1.0)^91^. FASTA files of chromosomes and genes (mRNA, proteins and non-coding RNA) and gff files for gene models and structural features (transposable element) can be downloaded from both the previously mentioned websites. Raw data (PacBio and Illumina reads) are available under the accession number PRJNA445774. RNA-seq data used for genome annotation are available under the following SRA accession numbers: SRP128461 for 91/100-5 leaves infected with blackspot and SRP133785 for *R.* *wichurana* and ‘Yesterday’ leaves infected with two powdery mildew pathotypes. Raw data of resequencing of the eight wild *Rosa* species are available under the SRA accession number SRP143586.

## Supplementary information

Supplementary Information Supplementary Methods, Supplementary References and Supplementary Figures 1–12

Reporting Checklist Reporting Summary file

Supplementary Table 1 Validation of the homozygosity of HapOB using ten microsatellites located on the 7 linkage groups

Supplementary Table 2 Summary of the PacBio and Illumina sequencing of HapOB

Supplementary Table 3 Prokaryotic gene prediction on the HapOB contigs

Supplementary Table 4 Details of the high-density SNP genetic maps from the OW progeny, obtained from a cross between R. *chinensis* ‘Old Blush’ (OB, female) and a hybrid of *R. wichurana* (W, male). Linkage maps for the maternal (A1 to A7) and paternal (B1 to B7) parents are provided, specifying the number of SNP per LG, the size (in cM), the number of unique loci per LG and the density of SNP/cM

Supplementary Table 5 Transposable element (TE) annotation in HapOB. A) Abundance of repetitive elements in the HapOB genome. Total number of elements per type and percentage relative to the total number of elements are shown. B) Comparison of TE in HapOB and *F. vesca* using automatic annotation with the REPET package: no manual curation step was done. C) Consensus library for TE annotation in HapOB genome. Each line represents a TE consensus genome family. TE consensus name: name of the consensus. Length (bp): TE consensus length in bp. Coverage (bp): cumulative genome coverage of this TE family in bp. Coverage (%): cumulative genome coverage of this TE family in percentage (calculated on 518515953 bp). Frags: number of TE fragments before the TEannot "long join procedure". fullLgthFrags: number of complete TE fragments before the TEannot ‘long join procedure’ (a full-length fragment represents only one fragment that covers 95% of the consensus). Copies: number of TE copies after the TEannot ‘long join procedure’ (a copy is a chain of fragments). fullLgthCopies: number of complete TE copies after the TEannot ‘long join procedure’ (a full-length copy is reconstructed by the join of fragmented multiple hits and all these fragments cover 95% of the consensus). meanId: mean identity calculated on the percentages of identity (between each copy with its TE consensus). sdId: standard deviation on the percentages of identities

Supplementary Table 6 SNP analysis in resequenced species within the genus *Rosa*. For each species, the detected SNP are classified according to their effect (HIGH (non synonymous change, splice sites), LOW (synonymous), MODERATE and MODIFIER (intron, intergenic)) or to their position in the genome (coding gene, intergenic)

Supplementary Table 7 Analysis of the elements inserted in A) *RoKSN* and in B) the *APETALA2/TOE* homologue, RC3G0243000. A) The *copia* element corresponding to the TE consensus RLC_denovoHm-B-G10244-Map11. This TE is inserted in four coding sequences, for which the gene function is described. For the full-length element, 3' and 5' Long Terminal Repeat (LTR) elements were aligned. The high similarity (around 100%) suggests a recent insertion. B) The inserted element in the *APETALA2/TOE* homologue corresponds a repeat element (consensus noCAT_denovoHM-B-R7962). The element is mainly inserted in non-coding regions (bar four insertions into introns of coding genes)

Supplementary Table 8 Detection of the *RoKSNnull* allele in *R.* *chinensis* 'Old Blush' using two different progenies. We studied the *RoKSN* segregation in two different progenies: (a) *R.* *chinensis* ‘Old Blush’ x R. wichurana hybrid (*RoKSNWT/RoKSNcopia*, OW progeny) and (b) *R. chinensis* ‘Old Blush’ x *R. moschata* (*RoKSNWT/RoKSNWT*, OM progeny). In both populations we observed unexpected allelic combinations, with individuals exhibiting only the *RoKSNWT* allele (47 individuals out of 152 in OW and 5 individuals out of 10 in OM). One hypothesis to explain these unexpected results is the existence of a null allele in *R. chinensis* ‘Old Blush’. In other words, our data suggests ‘Old Blush’ is not *RoKSNcopia/RoKSNcopia*, but *RoKSNcopia/RoKSNnull*

Supplementary Table 9 Precise location of the *DOUBLE FLOWER* locus using OW progenies with 151 individuals (OW151, in orange), with 260 individuals (OW260, in yellow), the HW (H190 x hybrid of *R. wichurana*, in purple), the 94/1 (in grey) and the YW (in blue) F1 progenies

Supplementary Table 10 Expression pattern during the floral development of the 41 candidate genes located in the *DOUBLE FLOWER* locus interval (see Supplementary Table 9). The expression value is expressed as the count number from RNASeq data obtained from Dubois et al. (2012): IFL for Floral Bud and Floral Meristem transition; IMO for Floral Meristem and Early Floral organs (Sepal, petal, stamens and carpel) developments; BFL for closed flower and OFT for open flower. The four most interesting candidate-genes according to their expression patterns are in blue and bold (see details in the text)

Supplementary Table 11 Association of the transposable element in *AP2/TOE* homologue with the double flower phenotype. We have developed a microsatellite marker, present in the *AP2* promoter region (2150bp downstream of the transposable element, see Supplementary Figure 7). 'Old Blush' (OB) has two alleles (180 and 166bp) as does *R. wichurana*, RW (186 and 169bp). By sequencing, we have shown that in OB the 166bp allele carries the transposable element, whereas the 180bp allele does not (Supplementary Figure 7). In RW, no transposable element is present. In the F1 progeny, all 154 simple-flower individuals possess the 180bp allele, whereas all 103 double-flower individuals possess the 166bp allele. As the microsatellite marker and the transposable element are in close proximity (2150bp), we can conclude that the 166bp allele / transposable element haplotype is associated with the double-flower phenotype

Supplementary Table 12 Rose cultivars of the association panel with data for prickle density and number of petals

Supplementary Table 13 Positioning and ordering of the contigs on the 7 pseudo-molecules. 196 contigs were anchored to the female and male genetic maps (more than one marker). The procedure for the positioning and ordering is given in Materials and Methods, and an example (Linkage group 5) is presented in Supplementary Figure 11. The average genetic position is presented for the male and female maps in cM. The contigs in red are those for which a manual analysis was performed, as described in Materials and Methods

## References

[1] Wang G (2007). A study on the history of Chinese roses from ancient works and images. Acta Hortic..

[2] Pliny (2013) Pine L'Ancience: Histoire naturelle (Schmit, S., Trans) *Bibliothèque de la Pléiade* No. 593 (Gallimard, Paris, 2013).

[3] Nybom H, Werlemark G (2017). Realizing the potential of health-promoting rosehips from dogroses (*Rosa* sect. *Caninae*). Curr. Bioact. Compd..

[4] Zhang J (2013). The diploid origins of allopolyploid rose species studied using single nucleotide polymorphism haplotypes flanking a microsatellite repeat. J. Hortic. Sci. Biotechnol..

[5] Ritz CM, Wisseman V (2011). Microsatellite analyses of artificial and spontaneous dogroses hybrids reveal the hybridogenic origin of *Rosa micrantha* by the contribution of unreduced gametes. J. Hered..

[6] Meng J, Fougère-Danezan M, Zhang LB, Li DZ, Yi TS (2011). Untangling the hybrid origin of the Chinese tea roses: evidence from DNA sequences of single-copy nuclear and chloroplast genes. Plant Syst. Evol..

[7] Wisseman V, Ritz CM (2005). The genus *Rosa* (Rosoideae, Rosaceae) revisited: molecular analysis of nrITS-1 and *atp*B*-rbc*L intergenic spacer (IGS) versus conventional taxonomy. Bot. J. Linna. Soc..

[8] Jian H (2012). Decaploidy in *Rosa praelucens* Byhouwer (Rosaceae) endemic to Zhongdian Plateau, Yunnan, China. Caryologia.

[9] Robert AV, Gladis T, Brumme H (2009). DNA amounts of roses (*R*osa L.) and their use in attributing ploidy levels. Plant Cell Rep..

[10] Bourke PM (2017). Partial preferential chromosome pairing is genotype dependent in tetraploid rose. Plant J..

[11] Herklotz V, Ritz CM (2017). Multiple and asymmetrical origin of polyploid dog rose hybrids (*Rosa* L. sect. *Caninae* (DC.) Ser.) involving unreduced gametes. Ann. Bot..

[12] Ritz CM, Köhnen I, Groth M, Theissen G, Wisseman V (2011). To be or not to be the odd one out—allele-specific transcription in pentaploid dogroses (*Rosa* L. sect. *Caninae* (DC.) Ser). BMC Plant Biol..

[13] Liorzou M (2016). Nineteenth century French rose (*Rosa* sp.) germplasm shows a shift over time from a European to an Asian genetic background. J. Exp. Bot..

[14] Nakamura N (2018). Genome structure of *Rosa multiflora*, a wild ancestor of cultivated roses. DNA Res..

[15] Wylie AP (1954). The history of garden roses. J. R. Hortic. Soc..

[16] Koren S (2017). Canu: scalable and accurate long-read assembly via adaptive k-mer weighting and repeat separation. Genome Res..

[17] Koning-Boucoiran CF (2015). Using RNA-seq to assemble a rose transcriptome with more than 13,000 full-length expressed genes and to develop the WagRhSNP 68k Axiom SNP array for rose (*Rosa* L.). Front. Plant Sci..

[18] Foissac S (2008). Genome annotation in plants and fungi: EuGene as a model platform. Curr. Bioinform..

[19] Jones P (2014). InterProScan 5: genome-scale protein function classification. Bioinformatics.

[20] Finn RD (2016). The Pfam protein families database: towards a more sustainable future. Nucleic Acids Res..

[21] Simao FA, Waterhouse RM, Ioannidis P, Kriventseva EV, Zdobnov EM (2015). BUSCO: assessing genome assembly and annotation completeness with single-copy orthologs. Bioinformatics.

[22] Edger PP (2018). Single-molecule sequencing and optical mapping yields an improved genome of woodland strawberry (*Fragaria vesca*) with chromosome-scale contiguity. GigaScience.

[23] Flutre T, Duprat E, Feuillet C, Quesneville H (2011). Considering transposable element diversification in de novo annotation approaches. PLoS ONE.

[24] Potter D (2007). Phylogeny and classification of Rosaceae. Plant Syst. Evol..

[25] Xiang Y (2017). Evolution of Rosaceae fruit types based on nuclear phylogeny in the context of geological times and genome duplication. Mol. Biol. Evol..

[26] Gar O (2011). An autotetraploid linkage map of rose (*Rosa hybrida*) validated using the strawberry (*Fragaria vesca*) genome sequence. PLoS ONE.

[27] Bruneau A, Starr JR, Joly S (2007). Phylogenetic relationships in the genus *Rosa*: new evidence from chloroplast DNA sequences and an appraisal of current knowledge. Syst. Bot..

[28] Fougère-Danezan M, Joly S, Bruneau A, Gao XF, Zhang LB (2015). Phylogeny and biogeography of wild roses with specific attention to polyploids. Ann. Bot..

[29] Fernández-Romero MD, Torres AM, Millán T, Cubero JI, Cabrera A (2001). Physical mapping of ribosomal DNA on several species of the subgenus *Rosa*. Theor. Appl. Genet..

[30] The 100 Tomato Genome Sequencing Consortium (2014). Exploring genetic variation in the tomato (*Solanum* section *Lycopersicon*) clade by whole-genome sequencing. Plant J..

[31] Duan N (2017). Genome re-sequencing reveals the history of apple and supports a two-stage model for fruit enlargement. Nat. Commun..

[32] Nguyen THN, Schulz D, Winkelmann T, Debener T (2017). Genetic dissection of adventitious shoot regeneration in roses by employing genome-wide association studies. Plant Cell Rep..

[33] Schulz DF (2016). Genome-wide association analysis of the anthocyanin and carotenoid contents of rose petals. Front. Plant Sci..

[34] Iwata H (2012). The *TFL1* homologue *KSN* is a regulator of continuous flowering in rose and strawberry. Plant J..

[35] Koskela EA (2012). Mutation in *TERMINAL FLOWER1* reverses the photoperiodic requirement for flowering in the wild strawberry *Fragaria vesca*. Plant Physiol..

[36] Shulaev V (2010). The genome of woodland strawberry (*Fragaria vesca*). Nat. Genet..

[37] Dubois A (2010). Tinkering with the C-function: a molecular frame for the selection of double flowers in cultivated roses. PLoS ONE.

[38] Roman H (2015). Genetic analysis of the flowering date and number of petals in rose. Tree Genet. Genomes.

[39] Shigyo M, Hasebe M, Ito M (2006). Molecular evolution of the AP2 subfamily. Gene.

[40] Bowman JL, Alvarez J, Weigel D, Meyerowitz EM, Smyth DR (1993). Control of flower development in *Arabidopsis thaliana* by APETALA1 and interacting genes. Development.

[41] Bowman JL, Smyth DR, Meyerowitz EM (1989). Genes directing flower development in *Arabidopsis*. Plant Cell.

[42] Jung JH, Lee S, Yun J, Lee M, Park CM (2014). The miR172 target TOE3 represses *AGAMOUS* expression during *Arabidopsis* floral patterning. Plant Sci..

[43] Zhang B, Wang L, Zeng L, Zhang C, Ma H (2015). *Arabidopsis* TOE proteins convey a photoperiodic signal to antagonize CONSTANS and regulate flowering time. Genes Dev..

[44] Bowman JL, Smyth DR, Meyerowitz EM (1991). Genetic interactions among floral homeotic genes of *Arabidopsis*. Development.

[45] Ó'Maoiléidigh DS, Graciet E, Wellmer F (2014). Gene networks controlling *Arabidopsis thaliana* flower development. New Phytol..

[46] Ashkani J, Rees DJG (2016). A comprehensive study of molecular evolution at the self-incompatibility locus of Rosaceae. J. Mol. Evol..

[47] Charlesworth D, Vekemans X, Castric V, Glemin S (2005). Plant self-incompatibility systems: a molecular evolutionary perspective. New Phytol..

[48] McClure B, Cruz-García F, Romero C (2011). Compatibility and incompatibility in S-RNase-based systems. Ann. Bot..

[49] Debener T (2010). Genetic and molecular analysis of key loci involved in self incompatibility and floral scent in roses. Acta Hortic..

[50] Mena-Ali JI, Stephenson AG (2007). Segregation analyses of partial self-incompatibility in self and cross progeny of *Solanum carolinense* reveal a leaky *S*-allele. Genetics.

[51] Kellogg AA, Branaman TJ, Jones NM, Little CZ, Swanson JD (2011). Morphological studies of developing *Rubus* prickles suggest that they are modified glandular trichomes. Botany.

[52] Pattanaik S, Patra B, Singh SK, Yuan L (2014). An overview of the gene regulatory network controlling trichome development in the model plant, *Arabidopsis*. Front. Plant Sci..

[53] Johnson CS, Kolevski B, Smyth DR (2002). *TRANSPARENT TESTA GLABRA2*, a trichome and seed coat development gene of *Arabidopsis*, encodes a WRKY transcription factor. Plant Cell.

[54] Magnard JL (2015). Biosynthesis of monoterpene scent compounds in roses. Science.

[55] Koning-Boucoiran CFS (2012). The mode of inheritance in tetraploid cut roses. Theor. Appl. Genet..

[56] Kyo M, Harada H (1986). Control of the developmental pathway of tobacco pollen in vitro. Planta.

[57] Hibrand-Saint Oyant L, Crespel L, Rajapakse S, Zhang L, Foucher F (2008). Genetic linkage maps of rose constructed with new microsatellite markers and locating QTL controlling flowering traits. Tree Genet. Genomes.

[58] Daccord N (2017). High-quality de novo assembly of the apple genome and methylome dynamics of early fruit development. Nat. Genet..

[59] Li H (2014). Toward better understanding of artifacts in variant calling from high-coverage samples. Bioinformatics.

[60] Walker BJ (2014). Pilon: an integrated tool for comprehensive microbial variant detection and genome assembly improvement. PLoS ONE.

[61] Noguchi H, Taniguchi T, Itoh T (2008). MetaGeneAnnotator: detecting species-specific patterns of ribosomal binding site for precise gene prediction in anonymous prokaryotic and phage genomes. DNA Res..

[62] Li H (2013). Aligning sequence reads, clone sequences and assembly contigs with BWA-MEM. arXiv.

[63] Hosseini Moghaddam H, Leus L, De Riek J, Van Huylenbroeck J, Van Bockstaele E (2012). Construction of a genetic linkage map with SSR, AFLP and morphological markers to locate QTLs controlling pathotype-specific powdery mildew resistance in diploid roses. Euphytica.

[64] Gitonga VW (2016). Inheritance and QTL analysis of the determinants of flower color in tetraploid cut roses. Mol. Breed..

[65] Yan Z, Dolstra O, Prins TW, Stam P, Visser PB (2006). Assessment of partial resistance to powdery mildew (*Podosphaera pannosa*) in a tetraploid rose population using a spore-suspension inoculation method. Eur. J. Plant Pathol..

[66] Bradbury PJ (2007). TASSEL: software for association mapping of complex traits in diverse samples. Bioinformatics.

[67] Kent WJ (2002). BLAT—the BLAST-like alignment tool. Genome Res..

[68] Benson G (1999). Tandem repeats finder: a program to analyze DNA sequences. Nucleic Acids Res..

[69] Novák P (2017). TAREAN: a computational tool for identification and characterization of satellite DNA from unassembled short reads. Nucleic Acids Res..

[70] Novak P, Neumann P, Pech J, Steinhaisl J, Macas J (2013). RepeatExplorer: a Galaxy-based web server for genome-wide characterization of eukaryotic repetitive elements from next-generation sequence reads. Bioinformatics.

[71] Langmead B, Salzberg SL (2012). Fast gapped-read alignment with Bowtie 2. Nat. Methods.

[72] Gu Z, Gu L, Eils R, Schlesner M, Brors B (2014). Circlize implements and enhances circular visualization in R. Bioinformatics.

[73] Gentleman RC (2004). Bioconductor: open software development for computational biology and bioinformatics. Genome Biol..

[74] Kirov I, Divashuk M, Van Laere K, Soloviev A, Khrustaleva L (2014). An easy “SteamDrop” method for high quality plant chromosome preparation. Mol. Cytogenet..

[75] Kirov IV, Van Laere K, Van Roy N, Khrustaleva LI (2016). Towards a FISH-based karyotype of *Rosa* L. (Rosaceae). Comp. Cytogenet..

[76] Kirov IV (2017). DRAWID: user-friendly java software for chromosome measurements and idiogram drawing. Comp. Cytogenet..

[77] Li H, Durbin R (2009). Fast and accurate short read alignment with Burrows–Wheeler transform. Bioinformatics.

[78] Li H (2011). A statistical framework for SNP calling, mutation discovery, association mapping and population genetical parameter estimation from sequencing data. Bioinformatics.

[79] McKenna A (2010). The Genome Analysis Toolkit: a MapReduce framework for analyzing next-generation DNA sequencing data. Genome Res..

[80] Cingolani P (2012). Using *Drosophila melanogaster* as a model for genotoxic chemical mutational studies with a new program, SnpSift. Front. Genet..

[81] Cingolani P (2012). A program for annotating and predicting the effects of single nucleotide polymorphisms, SnpEff: SNPs in the genome of *Drosophila melanogaster* strain w(1118); iso-2; iso-3. Fly.

[82] Stamatakis A (2014). RAxML version 8: a tool for phylogenetic analysis and post-analysis of large phylogenies. Bioinformatics.

[83] Lewis PO (2001). A likelihood approach to estimating phylogeny from discrete morphological character data. Syst. Biol..

[84] Du Mortier, B. C. *Notice sur un Nouveau Genre de Plantes: Hulthemia; Précédée d’un Aperçu sur la Classification des Roses* (Casterman, J., 1824).

[85] Lyons E (2008). Finding and comparing syntenic regions among *Arabidopsis* and the outgroups papaya, poplar, and grape: CoGe with rosids. Plant Physiol..

[86] Wang Y (2012). MCScanX: a toolkit for detection and evolutionary analysis of gene synteny and collinearity. Nucleic Acids Res..

[87] Krzywinski M (2009). Circos: an information aesthetic for comparative genomics. Genome Res..

[88] Bankevich A (2012). SPAdes: a new genome assembly algorithm and its applications to single-cell sequencing. J. Comput. Biol..

[89] Randoux M (2012). Gibberellins regulate the transcription of the continuous flowering regulator, RoKSN, a rose TFL1 homologue. J. Exp. Bot..

[90] Jung S (2014). The Genome Database for Rosaceae (GDR): year 10 update. Nucleic Acids Res..

