# Supplementary material for: A high-quality genome sequence of Rosa chinensis to elucidate ornamental traits
Source: Nat Plants. 2018 Jun 11;4(7):473–84. doi: 10.1038/s41477-018-0166-1 (PMC6786968; doi:10.1038/s41477-018-0166-1)
Supplement: Supplementary file 1 — Supplementary Methods, Supplementary References and Supplementary Figures 1–12 [file 41477_2018_166_MOESM1_ESM.pdf]

nature plants
SUPPLEMENTARY INFORMATION
https://doi.org/10.1038/s41477-018-0166-1
In the format provided by the authors and unedited.

A high-quality genome sequence of *Rosa chinensis* to elucidate ornamental traits

L. Hibrand Saint-Oyant[1], T. Ruttink[2], L. Hamama[1], I. Kirov[2,3], D. Lakhwani[1], N. N. Zhou[1], P. M. Bourke[4], N. Daccord[1], L. Leus[2], D. Schulz[5], H. Van de Geest[6], T. Hesselink[6], K. Van Laere[2], K. Debray[1], S. Balzergue[1], T. Thouroude[1], A. Chastellier[1], J. Jeauffre[1], L. Voisine[1], S. Gaillard[1], T. J. A. Borm[4], P. Arens[4], R. E. Voorrips[4], C. Maliepaard[4], E. Neu[5], M. Linde[5], M. C. Le Paslier[7], A. Bérard[7], R. Bounon[7], J. Clotault[1], N. Choisne[8], H. Quesneville[8], K. Kawamura[9], S. Aubourg[1], S. Sakr[1], M. J. M. Smulders[4], E. Schijlen[6], E. Bucher[1], T. Debener[5], J. De Riek[2] and F. Foucher[1]*

[1]IRHS, Agrocampus-Ouest, INRA, Université d'Angers, SFR 4207 QuaSaV, Beaucouzé, France. [2]ILVO, Flanders Research Institute for Agriculture, Fisheries and Food, Plant Sciences Unit, Melle, Belgium. [3]Russian State Agrarian University-Moscow Timiryazev Agricultural Academy, Moscow, Russia. [4]Plant Breeding, Wageningen University & Research, Wageningen, The Netherlands. [5]Leibniz Universität, Hannover, Germany. [6]Wageningen University & Research, Business Unit Bioscience, Wageningen, The Netherlands. [7]INRA, US 1279 EPGV, Université Paris-Saclay, Evry, France. [8]URGI, INRA, Université Paris-Saclay, Versailles, France. [9]Osaka Institute of Technology, Osaka, Japan. *e-mail: fabrice.foucher@inra.fr

© 2018 Macmillan Publishers Limited, part of Springer Nature. All rights reserved.

Supplementary Methods

Development of high density genetic maps

* F1 OW progeny.

Samples of young leaves were collected in spring 2014. Genomic DNA was isolated from nuclear extraction as previously described [1] and then purified using the Qiagen DNeasy 96 kit (Qiagen, MD, USA), according to the manufacturers recommendations. The Rose WagRhSNP 68k Axiom SNP array [2] was used for SNP genotyping, performed by the GENTYANE platform (INRA, Clermont Ferrand) according to Affymetrix recommendations.

SNP analysis was conducted using Genotyping Console and SNPolisher software (Affymetrix). Before constructing genetic maps, SNPs were filtered by excluding SNPs that were (*i*) monomorphic (AAxBB) and (*ii*) polymorphic between both parents (ABxAB), or (*iii*) with missing data. Consequently, only markers with segregation ABxAA, ABxBB, AAxAB, BBxAB were kept. We chose the best probe from the set of forward and reverse probes that were included on the array for each of the 68893 SNPs. The maximum marker distortion used to construct the genetic map was 1/3 – 2/3 corresponding to the distortion observed in the phenotypical segregation of the progenies with single versus double flowers [3]. The genetic map was built using the Kosambi function implemented in JoinMap 4.0 [4] using the "cross-pollinated" population type. The order of the markers was determined based on Maximum Likelihood and the grouping process used a maximum LOD of 10 to create the seven linkage groups (LG). To connect the new LG to the published reference rose genetic map [5], we added microsatellite markers in the first steps, these markers were then removed for the final analysis.

The genetic map was built using 151 individuals. Linkage analysis was performed using 5916 SNPs (ABxAA or ABxBB) for the female map and 2935 SNPs (AAxAB or BBxAB) for the male map. After the grouping process with a LOD of 10, 5635 (95%) and 2331 (79%) SNPs were selected to construct the seven LGs for the female and male genetic maps, respectively (Table 1). For the female map, the number of unique loci was 556 with a length of 482.8 cM corresponding to a map density of 0.87 marker/cM. The male genetic map contained 427 unique loci with a length of 474.1 cM and a map density of 0.9 marker/cM (Supplementary Table 4).

* F1 YW progeny

GBS markers were generated in an F1-population of 174 plants. 100 ng of genomic DNA was digested with *Eco*RI and *Msp*I, and ligated to TruSeq compatible GBS adapters with sample specific barcodes in a modified protocol from [6]. GBS-libraries were individually amplified with *Taq* 2x Mastermix NEB (Bioké), purified with MagNA magnetic beads, and pooled in equimolar amounts before sequencing on an Illumina HiSeq3000 instrument with 2x150bp at the Oklahoma Medical Research Facility (OMRF, USA). Reads were demultiplexed with GBSX 1.1.5 [7] allowing one mismatch in the barcodes. Sequence quality was checked with FastQC [8]. After QC of the raw reads, barcodes, restriction site remnants and adapter sequences were removed with Cutadapt [9] and FASTX-Toolkit 0.0.13 [10], and all reads were trimmed to a maximum length of 86bp to compensate for variable barcode lengths. Paired-end reads were overlapped using PEAR [11], and mapped onto the Old Blush reference genome sequence with default parameters of the BWA-mem algorithm in BWA 0.7.8 [12]. Alignments were sorted, indexed, and filtered on mapping quality 20 (q20) with SAMtools 1.2. [13]. Using 10 million quality filtered reads of the two parental lines, we first delineated GBS-stacks on the genome with the following criteria: length range 60-275 bp, average read depth 30-700, equal read depth ratio (0.75 to 1.33-fold) between both parents. SNPs were called with the Unified Genotyper implemented in Genome Analysis Toolkit (GATK) v.3.7 [14,15], and filtered with the following criteria: mean read depth DP10, minor allele count MAC4, SNP quality score q20, Genotype Quality score GQ30, min read depth per SNP per genotype DP10, bi-allelic SNPs. Finally, SNPs were selected from tags containing 1-3 neighbouring SNPs, with <10% missing-values across all 174 genotypes, and with a heterozygous genotype call in one parent and homozygous genotype call in the other parent. 2248 GBS-tags spread across the LGs of the 'Old Blush' reference genome with a total of 3622 SNPs segregating in the F1 were used to create the 'Yesterday' x *R. wichurana* genetic maps.

GBS data were combined with the AFLP and microsatellite data [16] for linkage mapping (unidirectional 'Yesterday' x *R. wichurana*, 139 F1 plants included). Joinmap 4.1 was applied to cluster the markers into 7 linkage groups; pwd-files with the pair wise recombination frequencies between markers and the corresponding LOD scores were exported. Marker ordering per linkage group was performed in SPSS v.23 using Multidimensional scaling (MDS, [17]). Pairwise recombination frequencies were taken as

distance input; LOD scores were used as weight factors. The unidimensional solution was accepted as the best ordering and compared for some linkage groups with map2 and map3 regression maps as generated by Joinmap.

Applying rather strict settings, 3622 GBS SNP markers were selected: 2320 segregating according to the <lmxll> Joinmap scheme; 1302 to the <nnxnp> scheme. Other segregation patterns were not retained. Combined with the previous set of Hosseini et al. (2012) [16] this yielded a set of 4575 markers. A LOD threshold range between 7 to 12 was applied in Joinmap to separate 7 potential linkage groups. A good correspondence with the pseudo-chromosome annotation to 'Old Blush' was observed; all SNP markers located on scaffolds currently assigned to Chr0 of HapOB were assigned to the 7 linkage groups of the YW map (Supplementary Figure 2b). For MDS mapping we compared both the use of LOD and LOD^2 values as weight parameters. Best correlation with map2 and map3 regression maps (Joinmap) was obtained by using LOD values as weights as recommended [17]. Per pseudochromosome the ordering of the linkage map was compared with the nucleotide position of markers on the physical sequence assembly. Specific recombination rates (kbp/cM) per chromosome were calculated (Supplementary Figure 1b). For all chromosomes a gradually increasing or decreasing trend was observed; nevertheless, different patterns were observed. Often two clouds of markers could be distinguished, which appeared to be caused by a poor integration of the maternal and paternal maps due to the limited number of allelic bridge markers in certain segments. This problem was resolved by separately calculating male and female maps.

* F1 K5 progeny

Tetraploid linkage maps of the K5 population, consisting of 151 unique individuals after screening and filtering, were generated following the methods described in Bourke et al. (2017) [18]. In brief, the population was genotyped using the WagRhSNP 68K Axiom SNP array, with marker dosage assigned using fitTetra [19]. Pairwise maximum likelihood recombination frequencies and LOD scores were calculated using polymapR package [20]. The code is available online through CRAN (https://CRAN.R-project.org/package=polymapR). Marker clustering by LOD score identified seven chromosomal linkage groups. Linkage group numbering was assigned through linkage analysis to previously-mapped SSR and AFLP markers [21]. Marker

ordering was performed using the MDSMap package [17], with outlying markers removed as recommended. The final integrated chromosomal maps consisting of 25,695 SNPs were validated through comparison to the *F. vesca* genome, with maps orientated according to the strawberry genome sequence.

KASP-assay for SNP validation

SNP markers for Kompetitive Allele Specific PCR (KASP) assays were designed by LGC Genomics (London, UK). Genotyping was performed using a StepOnePlus Real-Time PCR system (Applied Biosystems, USA) with 20 ng DNA, 5 µl KASP V4.0 Mastermix 96/384, High Rox, 0.14 µl KASP by Design Primer Mix in a final volume of 10 µl for each reaction. KASP thermocycling was done according to the manufacturer's standard protocol: Activation for 15 min at 94°C, followed by 10 cycles at 94°C for 20 s and 61°C for 1 min. (61°C decreasing 0.6°C per cycle to achieve final annealing temperature of 55°C) followed by 26 cycles at 94°C for 20 s and 55°C for 1 min. Reading of KASP genotyping reactions on the qPCR machine was performed in a final cycle at 30°C for 30 s. If fluorescence data did not form satisfactory clusters the conditions for additional cycles were 94°C for 20 s followed by 57°C for 1 min (up to 3 cycles). Genotypic data were analyzed with the StepOne™ Software v2.3 (Applied Biosystems, USA).

Development of a SCAR marker for the SI locus

The marker segregating at the SI locus was derived from a collection of genomic contigs generated by genomic sequencing of the diploid *R. multiflora* hybrid 88/124-46 via Illumina sequencing and subsequent assembly (data not shown). This collection of 27094 scaffolds was screened with 64 amino acid sequences of annotated S-RNAses from various *Prunus* species obtained from the NCBI database. One contig (contig no. 2308) with a significant hit to an S-RNAse gene also contained a predicted gene with similarity to an S-locus related F-Box protein. PCR primers (PrimerP9f 5´_CTTGCATTCAAGGTGCAGTC_3´ and Primer P9r 5´_CGGCTCTGGTGAAATAGTCC_3´) for the S-RNAse homolog were designed with the Primer3 software and flank the intron between exon 2 and 3 of the predicted S-RNAse sequence. Amplification conditions were: 94°C for 30s, 30 cycles of 94°C for 30s, 61°C for 30s and 72°C for 2min followed by a final amplification at 72°C for 10min.

Annotation of the rose genome

* Gene annotation

Previously, we developed a large data set of RNA-seq data from different tissues of 'Old Blush' [22]. In order to increase the mRNA diversity and the gene coverage, we also used RNA-seq libraries from four other genotypes with and without rose pathogens: *R. wichurana* and 'Yesterday' with two powdery mildew pathotypes and 'Pariser Charme' and 91/100-5 with black spot (Hannover) and all the *R. chinensis* EST and cDNA available in GenBank. RNA-seq reads were assembled using SOAPdenovo-Trans [23] before their integration in the annotation pipeline. The repeat regions of pseudo-chromosomes were masked using Red [24], LTRharvest [25] and BLASTX comparisons against Repbase [26]. The structural annotation of coding genes was performed using Eugene [27] by combining Gmap transcript mapping [28], similarities detected with plant proteomes and Swiss-Prot entries and *ab initio* prediction (Interpolated Markov Model and plant Weight Array Matric for donor and acceptor splicing sites). Furthermore, the Eugene pipeline included tRNAscan-SE [29], RNAmmer [30] and RfamScan [31] in order to annotate non-protein coding genes. The annotated genes were named using the following convention: RC (*Rosa chinensis*) followed by chromosome number, G, and gene number on the chromosome (steps of 100) going from top to bottom according to the linkage map e.g. RC4G0012800. The functional annotation of proteins was based on BLASTP results and InterProScan analysis [32] providing conserved motifs, gene family classification and Gene Ontology terms. Lastly, subcellular localization of proteins was predicted using TargetP [33].

All the structural and functional annotations and their supporting evidence are available through JBrowse [34] and can be downloaded (gff3 and fasta files) from the *Rosa* website (https://iris.angers.inra.fr/obh/).

* TE annotation

The TE*denovo* pipeline [35,36] from the REPET package v2.5 (https://urgi.versailles.inra.fr/Tools/REPET) was used to detect transposable elements (TEs) in genomic sequences to provide a consensus sequence for each TE family. For *Fragaria vesca*, the v2.0.a1 genome version was downloaded from GDR database (https://www.rosaceae.org/). First, the pseudo-chromosomes were deconstructed into 'virtual' contigs by removing stretches of more than 11 undefined bases (N's) to exclude

gaps in these sequences. We generated 564 'virtual' contigs with a N50 of 3 Mbp, with a total length of 513 Mbp for the *Rosa* genome and 13428 'virtual' contigs with a N50 of 28.6 Kb, with a total length of 197 Mbp for the *F. vesca* genome. For *Rosa* genome, a subset of 216 contigs (>300 kb) with at least five sequences per group was selected for TE detection (6020 contigs>10kb, with at least 3 sequences per group for *F. vesca*). After removing the redundancy and filtering consensus sequences (classified as SSR and Unclassified consensus constructed with less than 10 copies in the genome), a library of 7565 consensus sequences was obtained for *Rosa* genome (2534 consensus sequences for *F. vesca*) and classified according to structural and functional features based on characterised TEs from the RepBase21.01 database [37], a library of profiles from Pfam27.0 [38] and GyDB2.0 [39] specially formatted for REPET. Each TE library was used to annotate the TE copies in the whole genome using the TEannot pipeline [40] with default parameters resulting in a 65.8% TE coverage of the 518 Mbp rosa genome and 28.3% TE coverage of the 211 Mpb *F. vesca* genome.

A second TEannot iteration on the whole genomes was performed with a subset of consensus after filtering out those showing no full-length copy (i.e. copy covering more than 95% of the consensus sequence). For the *Rosa* genome, we selected 3901 consensus and 1543 for *F. vesca*. The second run of TEannot resulted in a TE coverage of 63.6% for *Rosa* and 27.4% for *Fragaria* showing that this process decreased the complexity of the consensus libraries (7565 to 3901 consensus sequences for *Rosa* and 2534 to 1543 consensus for *Fragaria*) without important loss in global TE coverage.

Finally, only for the *Rosa* genome, the library of 3901 consensus sequences was manually curated to re-classify some chimeric consensus sequences.

References :

1 Crespel, L. *et al.* Mapping of qualitative and quantitative phenotypic traits in Rosa using AFLP markers. *Theoretical and Applied Genetics* **105**, 1207-1214, doi:10.1007/s00122-002-1102-2 (2002).

2 Koning-Boucoiran, C. F. S. *et al.* Using RNA-Seq to assemble a rose transcriptome with more than 13,000 full-length expressed genes and to develop the WagRhSNP 68k Axiom SNP array for rose (*Rosa* L.). *Frontiers in Plant Science* **6**, 249, doi:10.3389/fpls.2015.00249 (2015).

3 Roman, H. *et al.* Genetic analysis of the flowering date and number of petals in rose. *Tree Genetics & Genomes* **2015**, 85, doi:10.1007/s11295-015-0906-6 (2015).

4 JoinMap(r) 4.0 sofware for the calculation of genetic linkage maps in experimental populations (Plant Research International, Wageningen, 2006).

5 Spiller, M. *et al.* Towards a unified genetic map for diploid roses. *Theor. Appl Genet* **122**, 489-500 (2011).
6 Poland, J. A., Brown, P. J., Sorrells, M. E. & Jannink, J.-L. Development of High-Density Genetic Maps for Barley and Wheat Using a Novel Two-Enzyme Genotyping-by-Sequencing Approach. *PLOS ONE* **7**, e32253, doi:10.1371/journal.pone.0032253 (2012).
7 Herten, K., Hestand, M. S., Vermeesch, J. R. & Van Houdt, J. K. J. GBSX: a toolkit for experimental design and demultiplexing genotyping by sequencing experiments. *BMC Bioinformatics* **16**, 73, doi:10.1186/s12859-015-0514-3 (2015).
8 Andrews, S. *FastQC A Quality Control tool for High Throughput Sequence Data*, <http://www.bioinformatics.babraham.ac.uk/projects/fastqc/> (
9 Martin, M. Cutadapt removes adapter sequences from high-throughput sequencing reads. *EMBnet.journal; Vol 17, No 1: Next Generation Sequencing Data Analysis* (2011).
10 FastX Toolkit (2010).
11 Zhang, J., Kobert, K., Flouri, T. & Stamatakis, A. PEAR: a fast and accurate Illumina Paired-End reAd mergeR. *Bioinformatics* **30**, 614-620, doi:10.1093/bioinformatics/btt593 (2014).
12 Li, H. & Durbin, R. Fast and accurate short read alignment with Burrows-Wheeler transform. *Bioinformatics* **25**, 1754-1760, doi:10.1093/bioinformatics/btp324 (2009).
13 Li, H. A statistical framework for SNP calling, mutation discovery, association mapping and population genetical parameter estimation from sequencing data. *Bioinformatics* **27**, 2987-2993, doi:10.1093/bioinformatics/btr509 (2011).
14 McKenna, A. *et al.* The Genome Analysis Toolkit: A MapReduce framework for analyzing next-generation DNA sequencing data. *Genome research* **20**, 1297-1303, doi:10.1101/gr.107524.110 (2010).
15 Van der Auwera, G. A. *et al.* From FastQ data to high confidence variant calls: the Genome Analysis Toolkit best practices pipeline. *Current protocols in bioinformatics / editoral board, Andreas D. Baxevanis ...[et al.]* **11**, 11.10.11-11.10.33, doi:10.1002/0471250953.bi1110s43 (2013).
16 Hosseini Moghaddam, H., Leus, L., De Riek, J., Van Huylenbroeck, J. & Van Bockstaele, E. Construction of a genetic linkage map with SSR, AFLP and morphological markers to locate QTLs controlling pathotype-specific powdery mildew resistance in diploid roses. *Euphytica* **184**, 413-427, doi:10.1007/s10681-011-0616-6 (2012).
17 Preedy, K. F. & Hackett, C. A. A rapid marker ordering approach for high-density genetic linkage maps in experimental autotetraploid populations using multidimensional scaling. *TAG. Theoretical and applied genetics. Theoretische und angewandte Genetik* **129**, 2117-2132, doi:10.1007/s00122-016-2761-8 (2016).
18 Bourke, P. M. *et al.* Partial preferential chromosome pairing is genotype dependent in tetraploid rose. *The Plant Journal* **90**, 330-343, doi:10.1111/tpj.13496 (2017).
19 Voorrips, R. E., Gort, G. & Vosman, B. Genotype calling in tetraploid species from bi-allelic marker data using mixture models. *BMC Bioinformatics* **12**, 172-172, doi:10.1186/1471-2105-12-172 (2011).
20 Bourke, P. *et al.* polymapR: linkage analysis and genetic map construction from F1 populations of outcrossing polyploids. *bioRxiv*, doi:10.1101/228817 (2017).

21 Koning-Boucoiran, C. F. S. *et al.* The mode of inheritance in tetraploid cut roses. *Theoretical and Applied Genetics* **125**, 591-607, doi:10.1007/s00122-012-1855-1 (2012).
22 Dubois, A. *et al.* Transcriptome database ressource and gene expression atlas for the rose. *BMC Genomics* **13**, 638 (2012).
23 Xie, Y. *et al.* SOAPdenovo-Trans: de novo transcriptome assembly with short RNA-Seq reads. *Bioinformatics* **30**, 1660-1666, doi:10.1093/bioinformatics/btu077 (2014).
24 Girgis, H. Z. Red: an intelligent, rapid, accurate tool for detecting repeats de-novo on the genomic scale. *BMC Bioinformatics* **16**, 227, doi:10.1186/s12859-015-0654-5 (2015).
25 Ellinghaus, D., Kurtz, S. & Willhoeft, U. LTRharvest, an efficient and flexible software for de novo detection of LTR retrotransposons. *BMC Bioinformatics* **9**, 18, doi:10.1186/1471-2105-9-18 (2008).
26 Bao, W., Kojima, K. K. & Kohany, O. Repbase Update, a database of repetitive elements in eukaryotic genomes. *Mobile DNA* **6**, 11, doi:10.1186/s13100-015-0041-9 (2015).
27 Foissac, S. *et al.* Genome Annotation in Plants and Fungi: EuGene as a Model Platform. *Current Bioinformatics* **3**, 87-97, doi:http://dx.doi.org/10.2174/157489308784340702 (2008).
28 Wu, T. D. & Watanabe, C. K. GMAP: a genomic mapping and alignment program for mRNA and EST sequences. *Bioinformatics* **21**, 1859-1875, doi:10.1093/bioinformatics/bti310 (2005).
29 Lowe, T. M. & Chan, P. P. tRNAscan-SE On-line: integrating search and context for analysis of transfer RNA genes. *Nucleic Acids Research* **44**, W54-W57, doi:10.1093/nar/gkw413 (2016).
30 Lagesen, K. *et al.* RNAmmer: consistent and rapid annotation of ribosomal RNA genes. *Nucleic Acids Research* **35**, 3100-3108, doi:10.1093/nar/gkm160 (2007).
31 Nawrocki, E. P. *et al.* Rfam 12.0: updates to the RNA families database. *Nucleic Acids Research* **43**, D130-D137, doi:10.1093/nar/gku1063 (2015).
32 Jones, P. *et al.* InterProScan 5: genome-scale protein function classification. *Bioinformatics* **30**, 1236-1240, doi:10.1093/bioinformatics/btu031 (2014).
33 Emanuelsson, O., Brunak, S., von Heijne, G. & Nielsen, H. Locating proteins in the cell using TargetP, SignalP and related tools. *Nature Protocols* **2**, 953-971, doi:10.1038/nprot.2007.131 (2007).
34 Skinner, M. E., Uzilov, A. V., Stein, L. D., Mungall, C. J. & Holmes, I. H. JBrowse: a next-generation genome browser. *Genome research* **19**, 1630-1638, doi:10.1101/gr.094607.109 (2009).
35 Flutre, T., Duprat, E., Feuillet, C. & Quesneville, H. Considering transposable element diversification in de novo annotation approaches. *PLoS One* **6**, e16526, doi:10.1371/journal.pone.0016526 (2011).
36 Hoede, C. *et al.* PASTEC: An Automatic Transposable Element Classification Tool. *PLOS ONE* **9**, e91929, doi:10.1371/journal.pone.0091929 (2014).
37 Jurka, J. *et al.* Repbase Update, a database of eukaryotic repetitive elements. *Cytogenetic and Genome Research* **110**, 462-467 (2005).
38 Finn, R. D. *et al.* The Pfam protein families database: towards a more sustainable future. *Nucleic Acids Research* **44**, D279-D285, doi:10.1093/nar/gkv1344 (2016).
39 Llorens, C. *et al.* The Gypsy Database (GyDB) of mobile genetic elements: release 2.0. *Nucleic Acids Research* **39**, D70-D74, doi:10.1093/nar/gkq1061 (2011).

40 Quesneville, H. *et al.* Combined Evidence Annotation of Transposable Elements in Genome Sequences. *PLOS Computational Biology* **1**, e22, doi:10.1371/journal.pcbi.0010022 (2005).

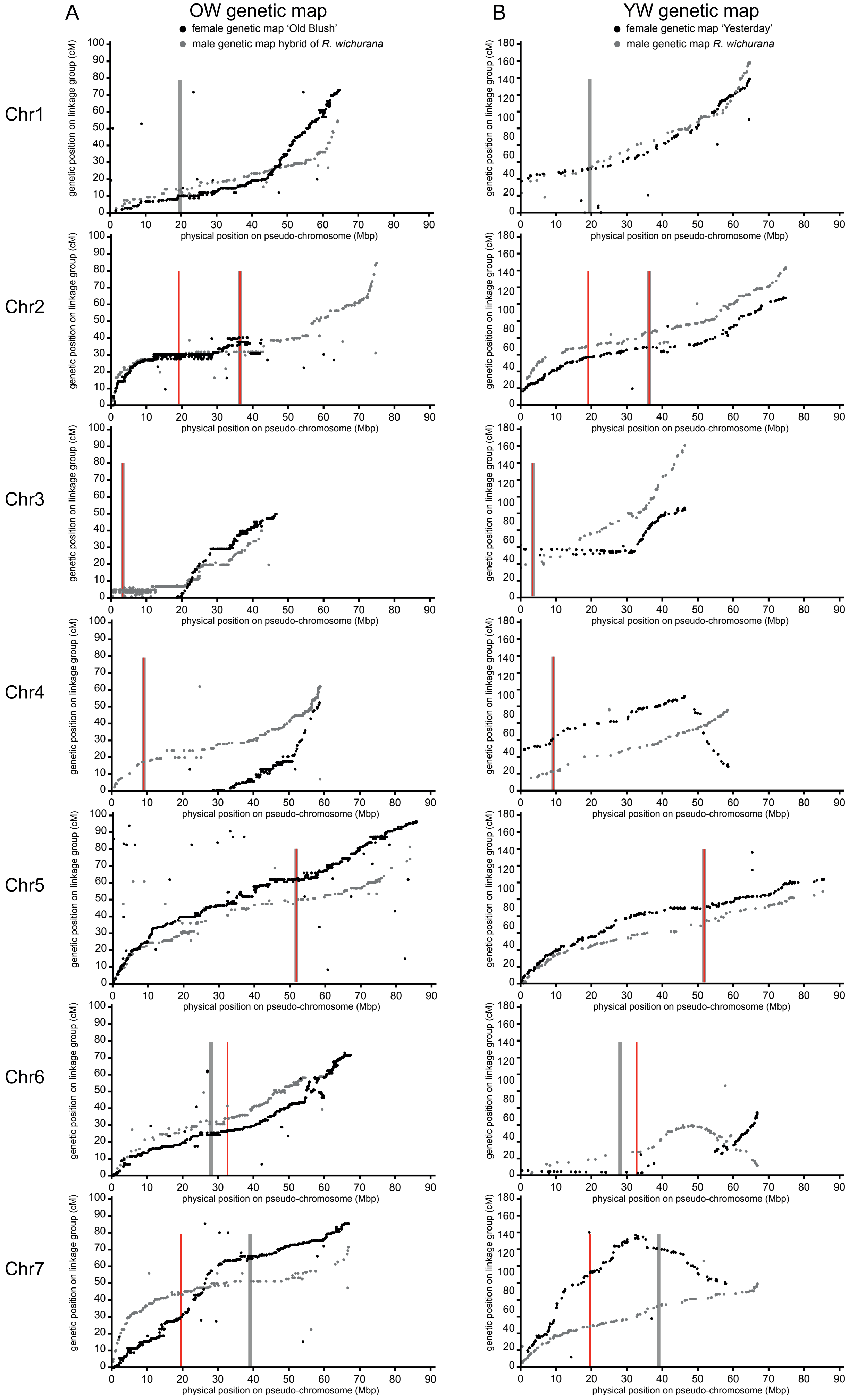

Supplementary Figure 1. Alignment of the OW and YW genetic maps to the pseudo-chromosomes of the HapOB physical sequence. Per mapping population, female (black circles) and male (gray circles) genetic maps are shown separately. Vertical bars show the location of OBC226 repeat sequences (red) and pericentromeric regions (gray).

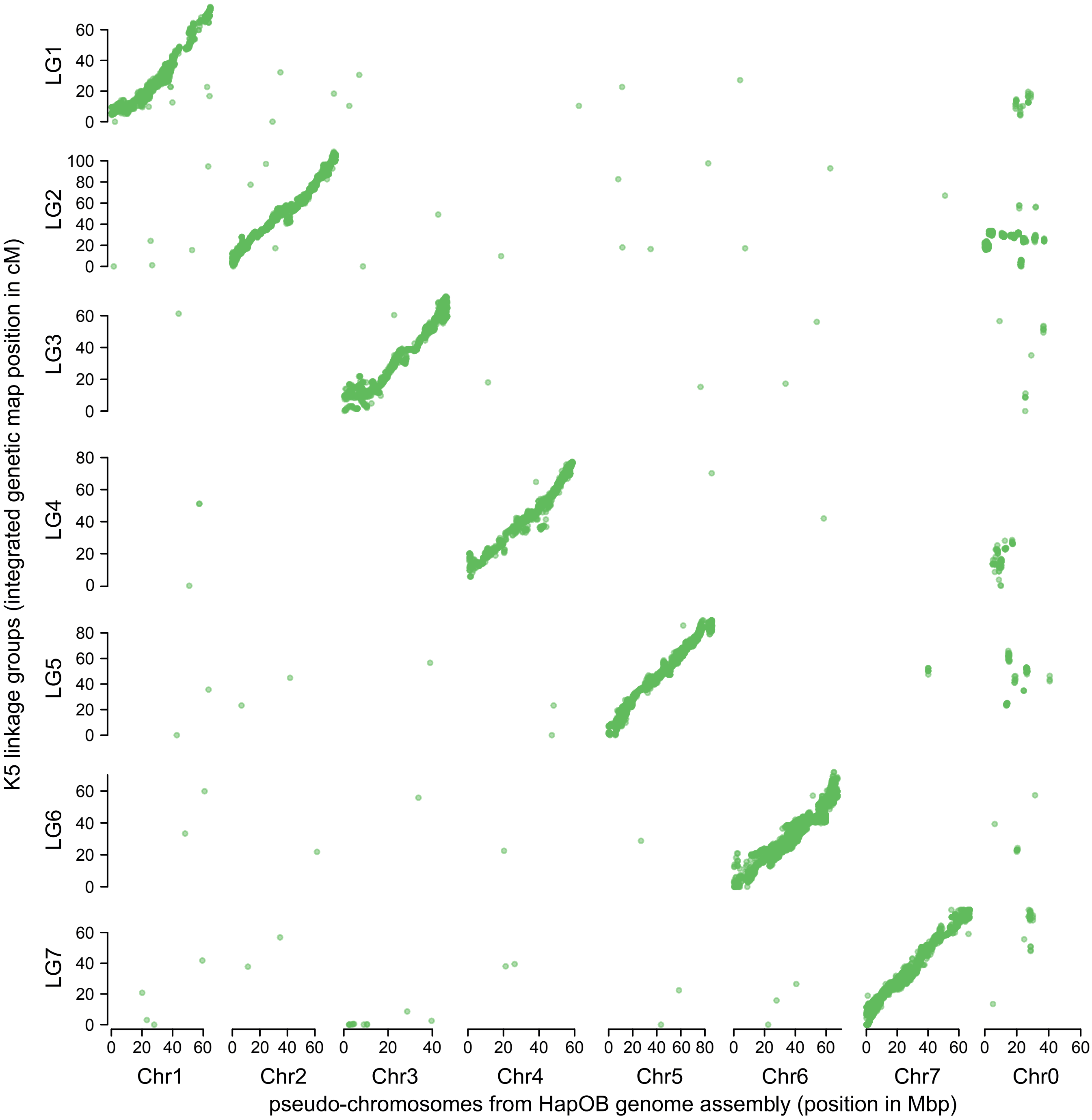

Supplementary Figure 2a: Alignment of the physical sequence of the seven pseudo-chromosomes of HapOB to the K5 integrated genetic map [10]. Several contigs that are currently assigned to Chr0 can be anchored to the different K5 linkage groups. LG3 of the K5 genetic map was inverted to be in the same orientation as the physical sequence of Chr3 of HapOB.

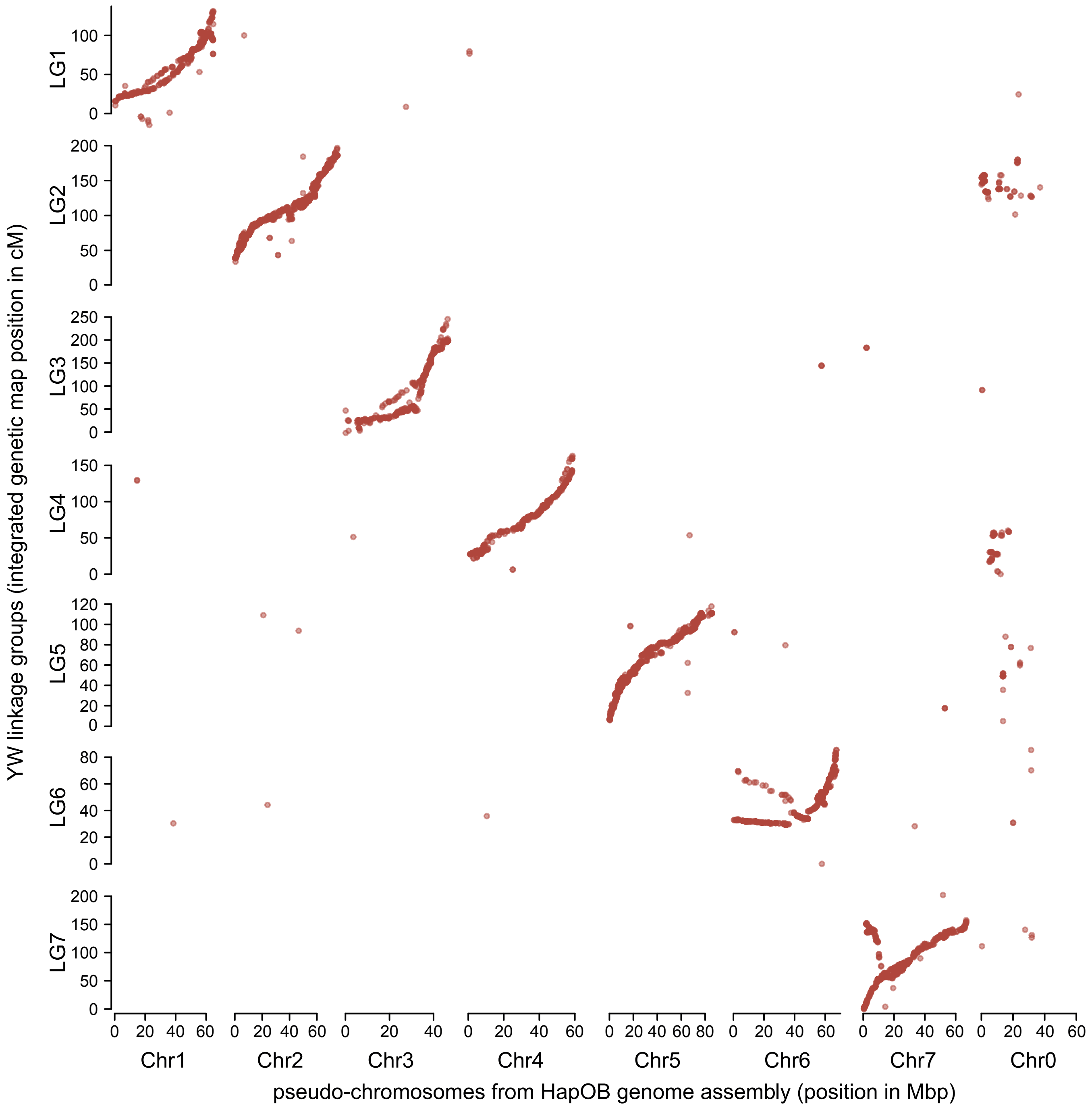

Supplementary Figure 2b: Alignment of the physical sequence of the seven pseudo-chromosomes of HapOB to the YW integrated genetic map. Several contigs that are currently assigned to Chr0 can be anchored to the different YW linkage groups.

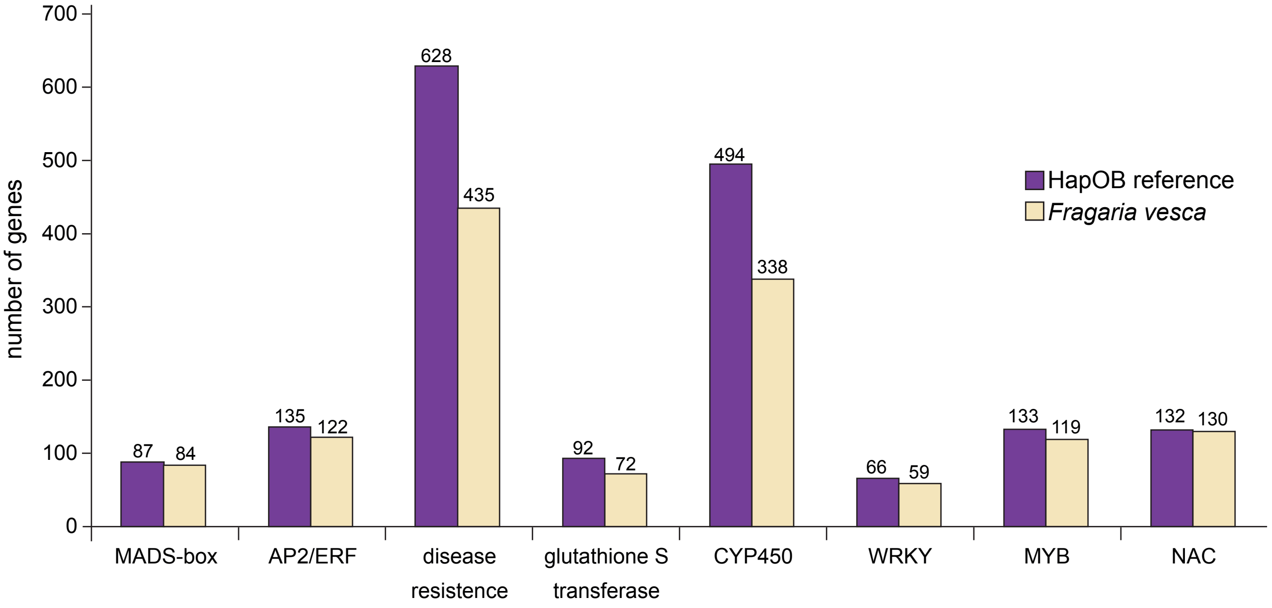

Supplementary Figure 3. Number of predicted proteins for different protein families in the HapOB reference genome and in *Fragaria vesca*.

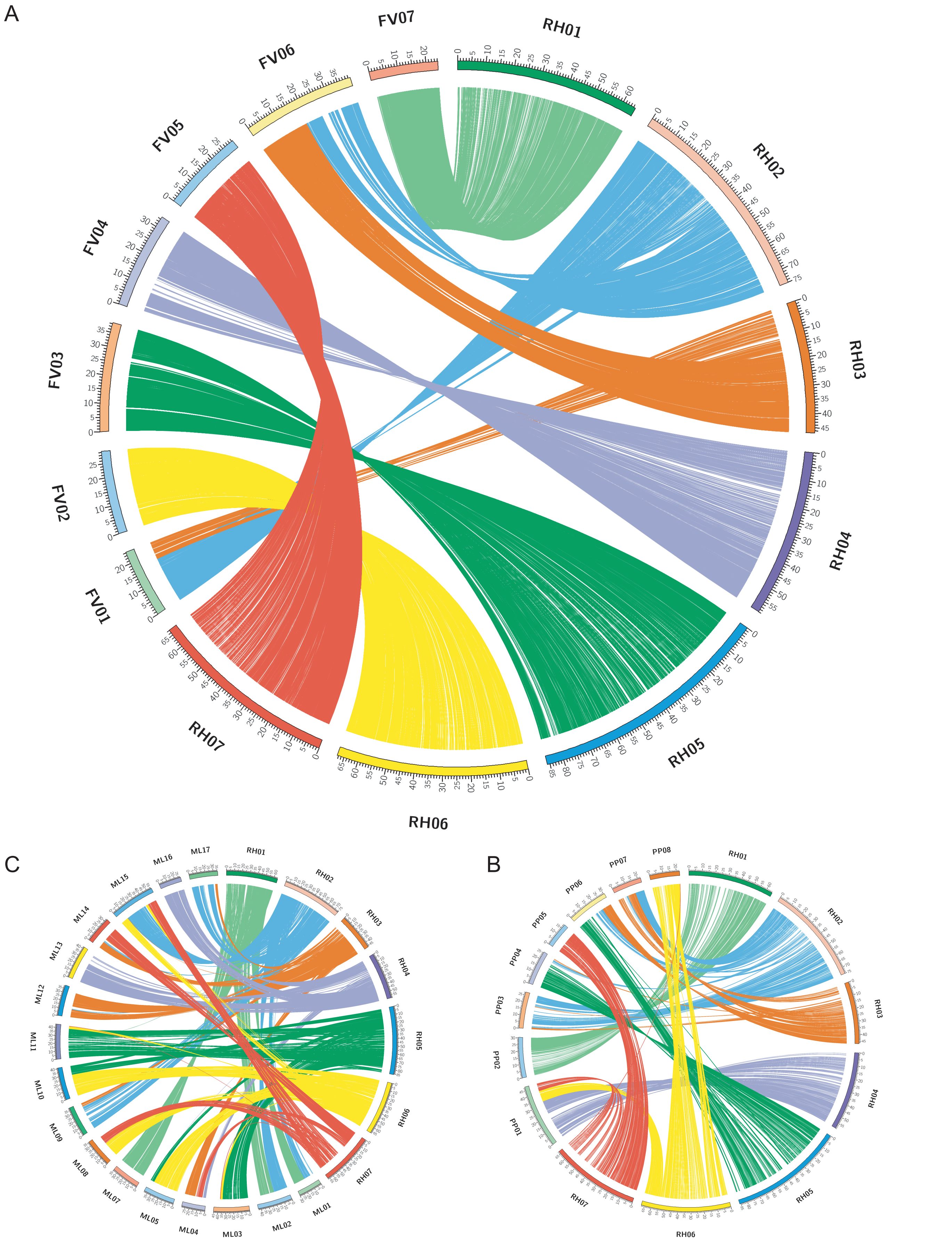

Supplementary Figure 4. Synteny analysis between the HapOB genome (RH) and A) the woodland strawberry genome (*Fragaria vesca*, FV), B) the apple genome (*Malus,* ML) and C) the plum genome (*Prunus persica,* PP), respectively. The numbering of the rose chromosomes follows Spiller et al., (2011).

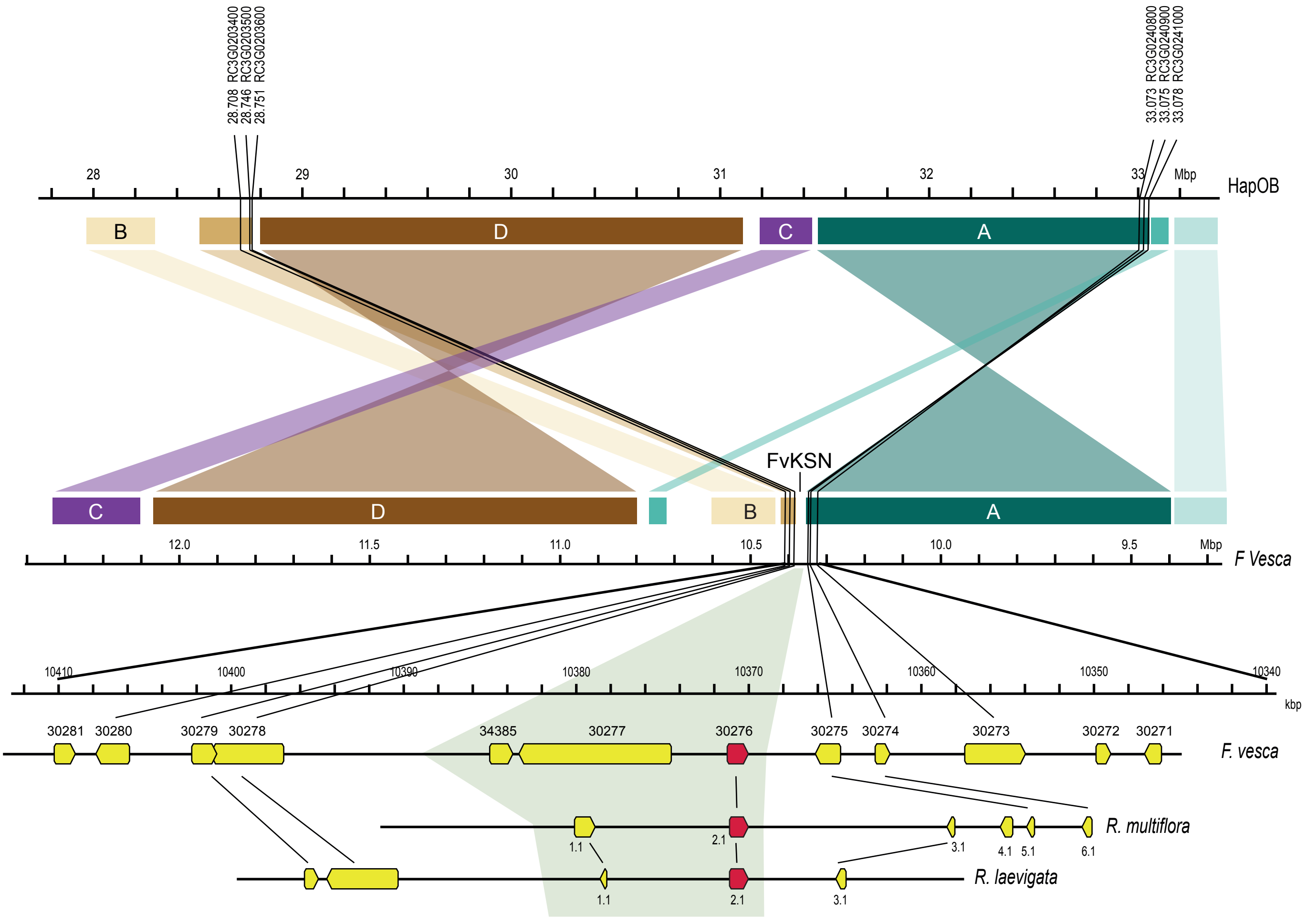

Supplementary Figure 5. Analysis of the *RoKSN*^{null} allele in HapOB. The upper part of the figure represents the macrosynteny analysis between the *CONTINUOUS FLOWERING* locus in HapOB and *Fragaria vesca*. Large segmental rearrangements were detected between conserved blocks (A, B, C and D) and no *RoKSN* homolog is present in HapOB. The lower part represents the microsynteny at the *FvKSN* locus between *F. vesca* and once-flowering roses (*R. multiflora* and *R. laevigata*). In these once-flowering roses, the synteny is conserved (no rearrangement) and the *RoKSN* gene is present (shaded area in lower panel). The *KSN* homologues are shown in red (gene 30276 is *FvKSN*). Other orthologous genes are connected by black lines.

```
            1        10        20        30        40        50        60        70
Consensus   MEEANLPEEIVVLILSWLPVKSLLRFTCVSKXFHFIILSNPKFAKSQFQAARERKTLDQRLLYNTTVPPLESL
Identity
1. HapOB    MEEANLPEEIVVLILSWLPVKSLLRFTCVSKSFHFIILSNPKFAKSQFQAARERKTLDQRLLYNTTVPPLESL
2. OB2      MEEANLPEEIVVLILSWLPVKSLLRFTCVSKRFHFIILSNPKFAKSQFQAARERKTLDQRLLYNTTVPPLESL

                  80        90       100       110       120       130       140
Consensus   DLETLPLFGDSSYLERRLLYSKIAPRLESLHLETTSFGDRSSVRKLQFPFQPPVGRLSLLGSCNGIVFLAFDE
Identity
1. HapOB    DLETLPLFGDSSYLERRLLYSKIAPRLESLHLETTSFGDRSSVRKLQFPFQPPVGRLSLLGSCNGIVFLAFDE
2. OB2      DLETLPLFGDSSYLERRLLYSKIAPRLESLHLETTSFGDRSSVRKLQFPFQPPVGRLSLLGSCNGIVFLAFDE

               150       160       170       180       190       200       210
Consensus   RVFYIWNPSTGFFRKLPDPGSLCDQRFLFNYGVGYLSATDDYKIFIATDVYAIFSSRAQAWKKLEVYSESPPS
Identity
1. HapOB    RVFYIWNPSTGFFRKLPDPGSLCDQRFLFNYGVGYLSATDDYKIFIATDVYAIFSSRAQAWKKLEVYSESPPS
2. OB2      RVFYIWNPSTGFFRKLPDPGSLCDQRFLFNYGVGYLSATDDYKIFIATDVYAIFSSRAQAWKKLEVYSESPPS

            220       230       240       250       260       270       280       290
Consensus   XRGTLLNEALHWLNQENGIVAFDLAQEKFSKMTMPNFDERSLNQFGYLGVSAEGCLSFALSLWDAHDCIQVWV
Identity
1. HapOB    YRGTLLNEALHWLNQENGIVAFDLAQEKFSKMTMPNFDERSLNQFGYLGVSAEGCLSFALSLWDAHDCIQVWV
2. OB2      FRGTLLNEALHWLNQENGIVAFDLAQEKFSKMTMPNFDERSLNQFGYLGVSAEGCLSFALSLWDAHDCIQVWV

                   300       310       320       330       340       350       360
Consensus   MKEYGVHDSWTKLYNFRFLDPPEGMWSFSRVLVLETSIVAHICTMKEVDNDCITDVKGLIKILHKEEEKCGEY
Identity
1. HapOB    MKEYGVHDSWTKLYNFRFLDPPEGMWSFSRVLVLETSIVAHICTMKEVDNDCITDVKGLIKILHKEEEKCGEY
2. OB2      MKEYGVHDSWTKLYNFRFLDPPEGMWSFSRVLVLETSIVAHICTMKEVDNDCITDVKGLIKILHKEEEKCGEY

                370       380   386
Consensus   VIEGFELKMIRYQESLCRIDD
Identity
1. HapOB    VIEGFELKMIRYQESLCRIDD
2. OB2      VIEGFELKMIRYQESL
```

Supplementary Figure 6a. Amino acid alignments of the two alleles of an F-Box protein encoded by the Old Blush gene RC3G0245100 at the *DOUBLE-FLOWER* locus. 1. HapOB: the allele present in the HapOB reference sequence; 2. OB2: the second allele of ‘Old Blush’.

```
           1        10        20        30        40        50        60        70        80        90
Consensus  MRVLV ILGYTSSSISTPPNPNGNRSTKPYKPPIPKLSPSKSSPPISTLHSRPPPLFAGRWDPHNTHLSYFADLASKLARDGKLDDFAMIAE
Identity

1. HapOB   MRVLV ILGYTSSSISTPPNPNGNRSTKPYKPPIPKLSPSKSSPPISTLHSRPPPLFAGRWDPHNTHLSYFADLASKLARDGKLDDFAMIAE
2. OB2     MRVLV ILGYTSSSISTPPNPNGNRSTKPYKPPIPKLSPSKSSPPISTLHSRPPPLFAGRWDPHNTHLSYFADLASKLARDGKLDDFAMIAE

                   100       110       120       130       140       150       160       170       180
Consensus  SVVLSGVKPSQFAAALKVDMVARGISGFLKEGKVRRVLEVLTKVDELGVRPLELFDGYAMELLGGHCHRLLKFKXVQELVELMEVLSGLHF
Identity

1. HapOB   SVVLSGVKPSQFAAALKVDMVARGISGFLKEGKVRRVLEVLTKVDELGVRPLELFDGYAMELLGGHCHRLLKFKRVQELVELMEVLSGLHF
2. OB2     SVVLSGVKPSQFAAALKVDMVARGISGFLKEGKVRRVXEVLTKVDELGVRPLELFDGYAMELLGGHCHRLLKFKQVQELVELMEVLSGLHF

                  190       200       210       220       230       240       250       260       270
Consensus  PIKELVEPSEVIKTCVDKRSPKLAIRYACIFPHAHILFCNIMNEFGKRRALASALTAYEASKZKLSGSNMYIYRTIIDVCGVCKDYMKSRY
Identity

1. HapOB   PIKELVEPSEVIKTCVDKRSPKLAIRYACIFPHAHILFCNIMNEFGKRRALASALTAYEASKEKLSGSNMYIYRTIIDVCGVCKDYMKSRY
2. OB2     PIKELVEPSEVIKTCVDKRSPKLAIRYACIFPHAHILFCNIMNEFGKRRALASALTAYEASKQKLSGSNMYIYRTIIDVCGVCKDYMKSRY

                280       290       300       310       320       330       340       350       360
Consensus  IYEDLLKQKVIPNIYVFNSLMNVNSHDLSYTFHVYKSMQNLGVKADLACYNILLKACCLAGRVDLAQDIYREVQHLESTGVLKLDVFTYST
Identity

1. HapOB   IYEDLLKQKVIPNIYVFNSLMNVNSHDLSYTFHVYKSMQNLGVKADLACYNILLKACCLAGRVDLAQDIYREVQHLESTGVLKLDVFTYST
2. OB2     IYEDLLKQKVIPNIYVFNSLMNVNSHDLSYTFHVYKSMQNLGVKADLACYNILLKACCLAGRVDLAQDIYREVQHLESTGVLKLDVFTYST

              370       380       390       400       410       420       430       440       450
Consensus  IVKVFSDAKMWHMALNVKEDMLAAGVMPNTITWSSLISACANAGLVDKAIQLFEEMLLVGCEPNSQCFNILLHACVEACQYDRAFRLFHSF
Identity

1. HapOB   IVKVFSDAKMWHMALNVKEDMLAAGVMPNTITWSSLISACANAGLVDKAIQLFEEMLLVGCEPNSQCFNILLHACVEACQYDRAFRLFHSF
2. OB2     IVKVFSDAKMWHMALNVKEDMXAAGVMPNTITWSSLISACANAGLVDKAIQLFEEMLLXGCEPNSQCFNILLHACVEACQYDRAFRLFHSF

            460       470       480       490       500       510       520       530       540
Consensus  KSNKVQETFGKNYKGXSFKPTTTTYNTLMKACGSDYYHARALMDEMKTVGLFPNQITWSILADICGSSGNVEGALQILKSMRVAGIQPDVV
Identity

1. HapOB   KSNKVQETFGKNYKGLSFKPTTTTYNTLMKACGSDYYHARALMDEMKTVGLFPNQITWSILADICGSSGNVEGALQILKSMRVAGIQPDVV
2. OB2     KSNKVQETFGKNYKGRSFKPTTTTYNTLMKACGSDYYHARALMDEMKTVGLFPNQITWSILADICGSSGNVEGALQILKSMRVAGIQPDVV

            550       560       570       580       590       600       610       620       630
Consensus  AYTTAIKICVESENLDLALSLFAEMKKYQIHPNLVTYNTLLRARSRYGSVSEVQQCLAIYQDMRKAGYKPNDYYLEQLIEEWCEGVIQDSS
Identity

1. HapOB   AYTTAIKICVESENLDLALSLFAEMKKYQIHPNLVTYNTLLRARSRYGSVSEVQQCLAIYQDMRKAGYKPNDYYLEQLIEEWCEGVIQDSS
2. OB2     AYTTAIKICVESENLDLALSLFAEMKKYQIHPNLVTYNTLLRARSRYGSVSEVQQCLAIYQDMRKAGYKPNDYYLEQLIEEWCEGVIQDSS

            640       650       660       670       680       690       700       710       720
Consensus  PKQGEFSYWDKAGIGRPGSVLLEKVAEHLQQHIADTLAVDLQGLTKVEARIVVLAVLRMIKENYTLGDSVKDDMLIMVGVHDEVDGGSTAH
Identity

1. HapOB   PKQGEFSYWDKAGIGRPGSVLLEKVAEHLQQHIADTLAVDLQGLTKVEARIVVLAVLRMIKENYTLGDSVKDDMLIMVGVHDEVDGGSTAH
2. OB2     PKQGEFSYWDKAGIGRPGSVLLEKVAEHLQQHIADTLAVDLQGLTKVEARIVVLAVLRMIKENYTLGDSVKDDMLIMVGVHDEVDGGSTAH

           730       740       750       760       770       780       790       800       810      818
Consensus  NLEVIDAITKLLQDELGLKVLSTVPKVALDTTIVSENNIDSDQTLEEKPVRNELHSELIYSTRRPVVLERLXVSRKSLQQWLRKRSSLRR
Identity

1. HapOB   NLEVIDAITKLLQDELGLKVLSTVPKVALDTTIVSENNIDSDQTLEEKPVRNELHSELIYSTRRPVVLERLKVSRKSLQQWLRKRSSLRR
2. OB2     NLEVIDAITKLLQDELGLKVLSTVPKVALDTTIVSENNIDSDQTLEEKPVRNELHSELIYSTRRPVVLERLNVSRKSLQQWLRKRSSLRR
```

Supplementary Figure 6b. Amino acid alignments of the two alleles of a tetratricopeptide repeat (TPR)-like family protein encoded by the Old Blush gene RC3G0243500 at the *DOUBLE-FLOWER* locus. 1. HapOB: the allele present in the HapOB reference sequence; 2. OB2: the second allele of 'Old Blush'.

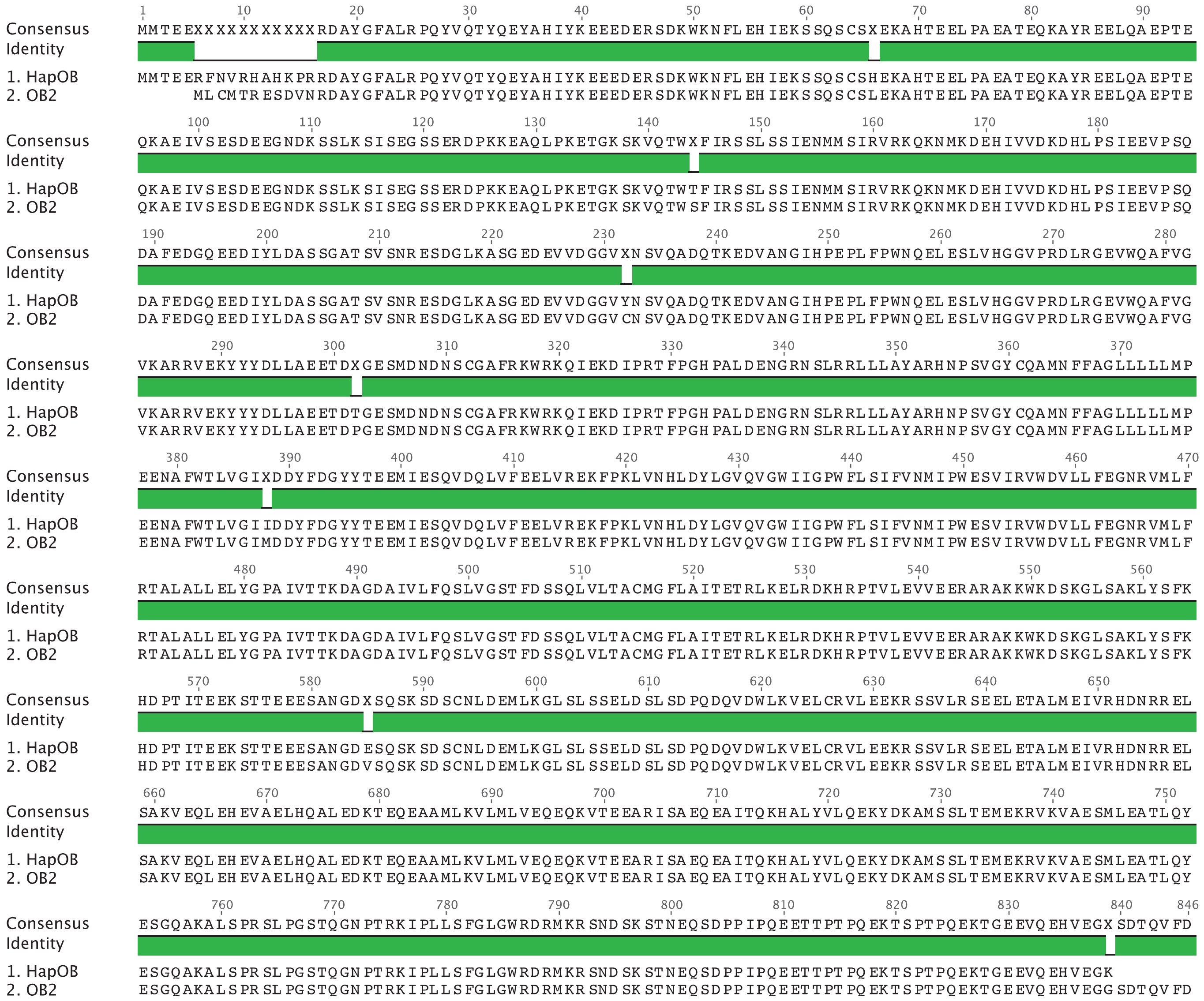

Supplementary Figure 6c. Amino acid alignments of the two alleles of a protein with high similarity to the Ypt/Rab-GAP domain of the gyp1p super family, encoded by the Old Blush gene RC3G0245000 at the *DOUBLE-FLOWER* locus. 1. HapOB: the allele present in the HapOB reference sequence; 2. OB2: the second allele of 'Old Blush'.

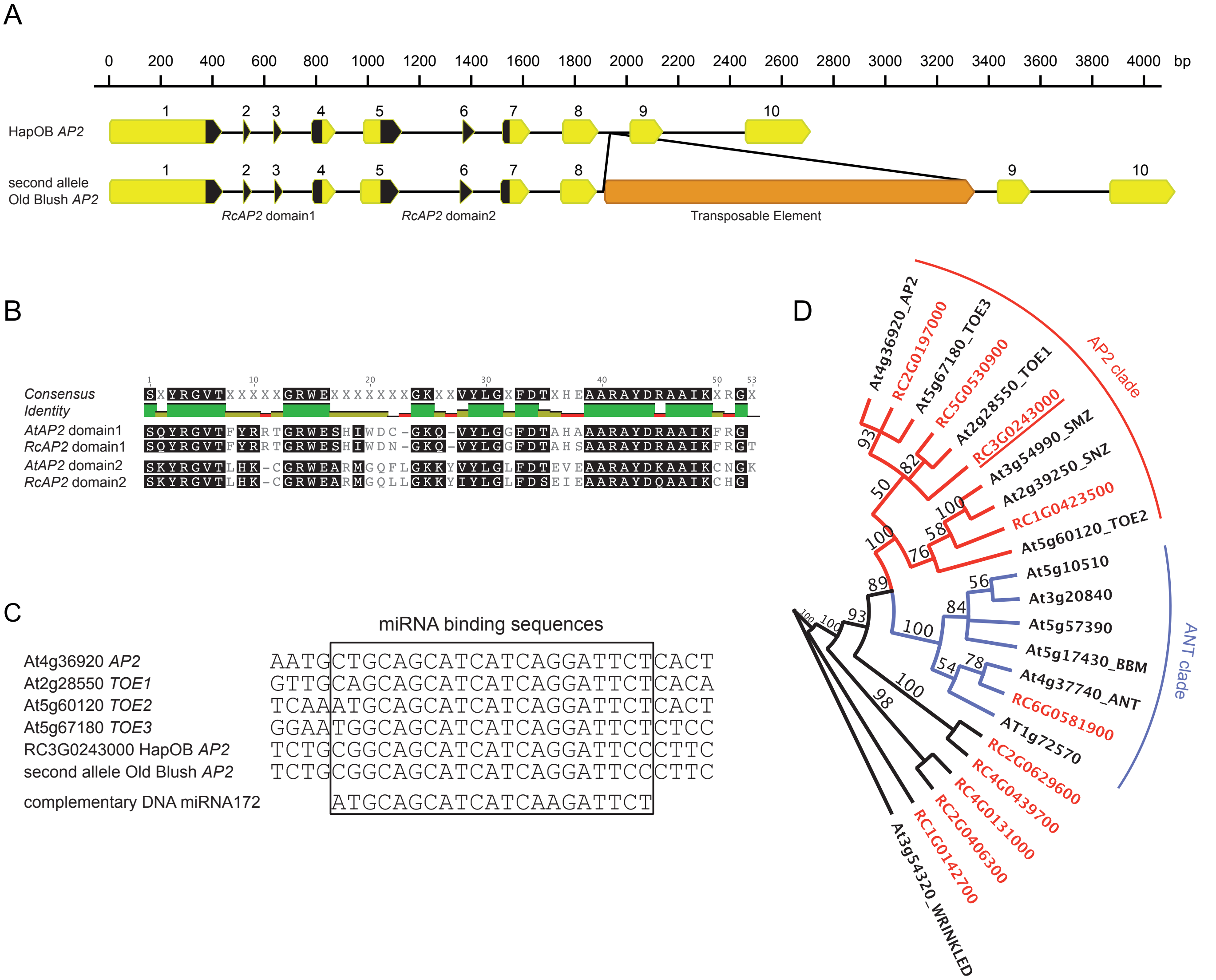

Supplementary Figure 7. Analysis of the two *APETALA2/TOE* alleles in 'Old Blush'.

A) genomic organisation of the two alleles of RC3G0243000. The gene contains 10 exons (numbered yellow boxes show CDS) and 9 introns. The main difference between both alleles is the insertion of a large transposable element (orange box, 1426 bp) in intron 8 of one Old Blush *AP2* allele.

B) conservation of two AP2 domains in APETALA2 of *Arabidopsis thaliana* (*At*) and rose (*Rc*).

C) conservation of the *miRNA172* binding sequences in the *Arabidopsis* AP2 clade genes (*AP2*, *TOE1*, *TOE2* and *TOE3*) and the two alleles of the *APETALA2* homolog in rose.

D) Phylogenetic analysis of the AP2 and ANT clades of the APETALA2/TOE protein subfamily of rose (in red) and *A. thaliana* (in black). The *Arabidopsis* proteins are numbered according to TAIR nomenclature (http://www.arabidopsis.org). The best candidate gene for double flower in rose (RC3G0243000) is underlined.

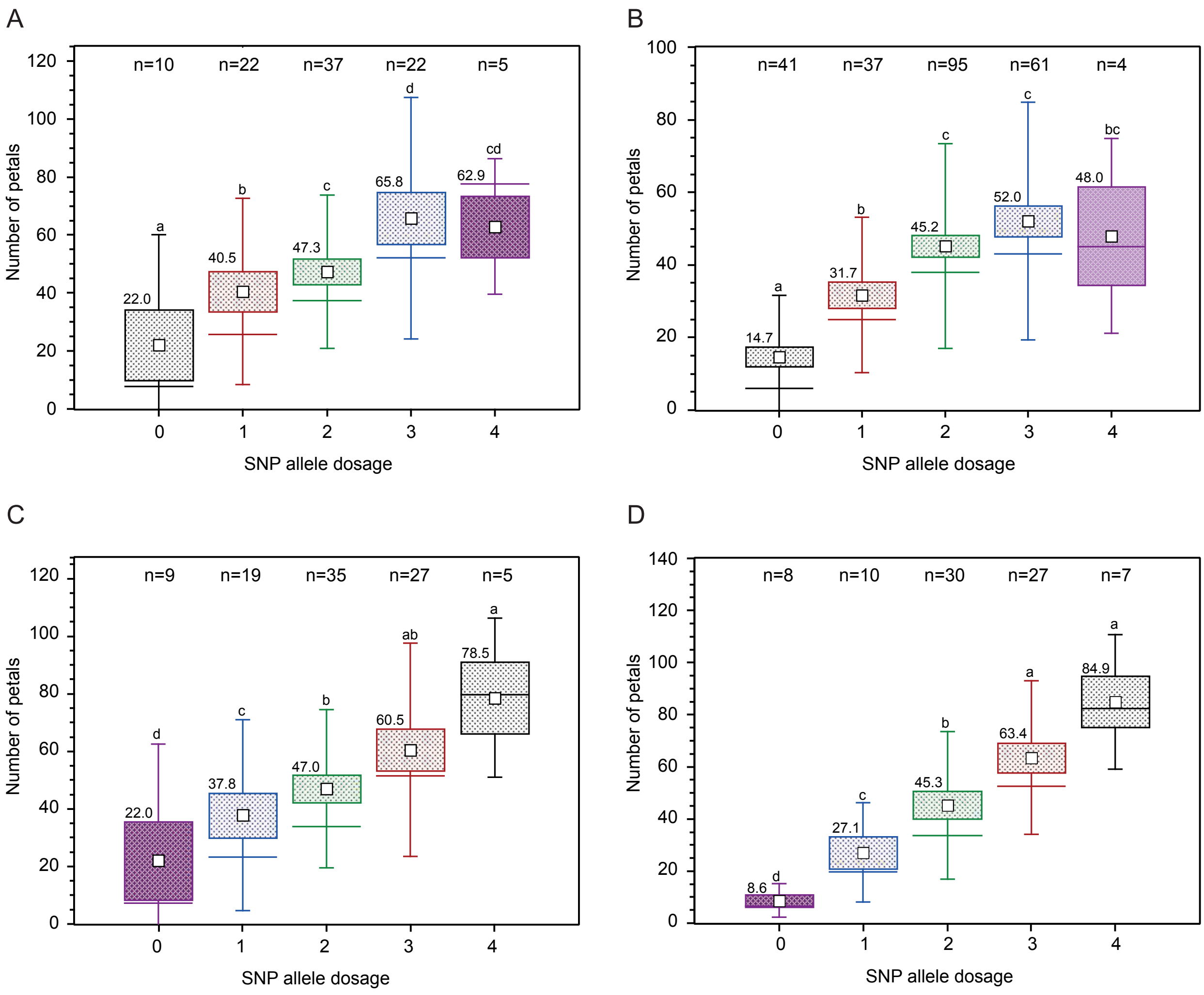

Supplementary Figure 8. Validation of SNP markers for petal number. A-B) marker RhK5_4359_382 (at position 33.55 Mbp) in an association panel of 96 cultivars (A), and in 238 independent tetraploid rose cultivars (B). C) marker RhK5_14942 (at Chr3 position 33.24 Mbp) in an association panel of 96 cultivars. D) marker RhMCRND_760_1045 (at Chr3 position 33.21 Mbp) in an association panel of 96 cultivars. Numbers on x-axis show allele dosage for the four marker classes (0 and 4 for the alternative homozygotes, and 1-3 for the heterozygotes). Per allele dosage group, the number of individuals (n) is given on top; groups that are significantly different at $p \leq 0.05$ are indicated by letters above the whiskers (pairwise comparison with a Mann-Whitney U test (two sided)). The mean is represented by small white squares, and the median by the horizontal line. Mean values are given above the box. Whiskers represent the standard deviation and box size the standard error.

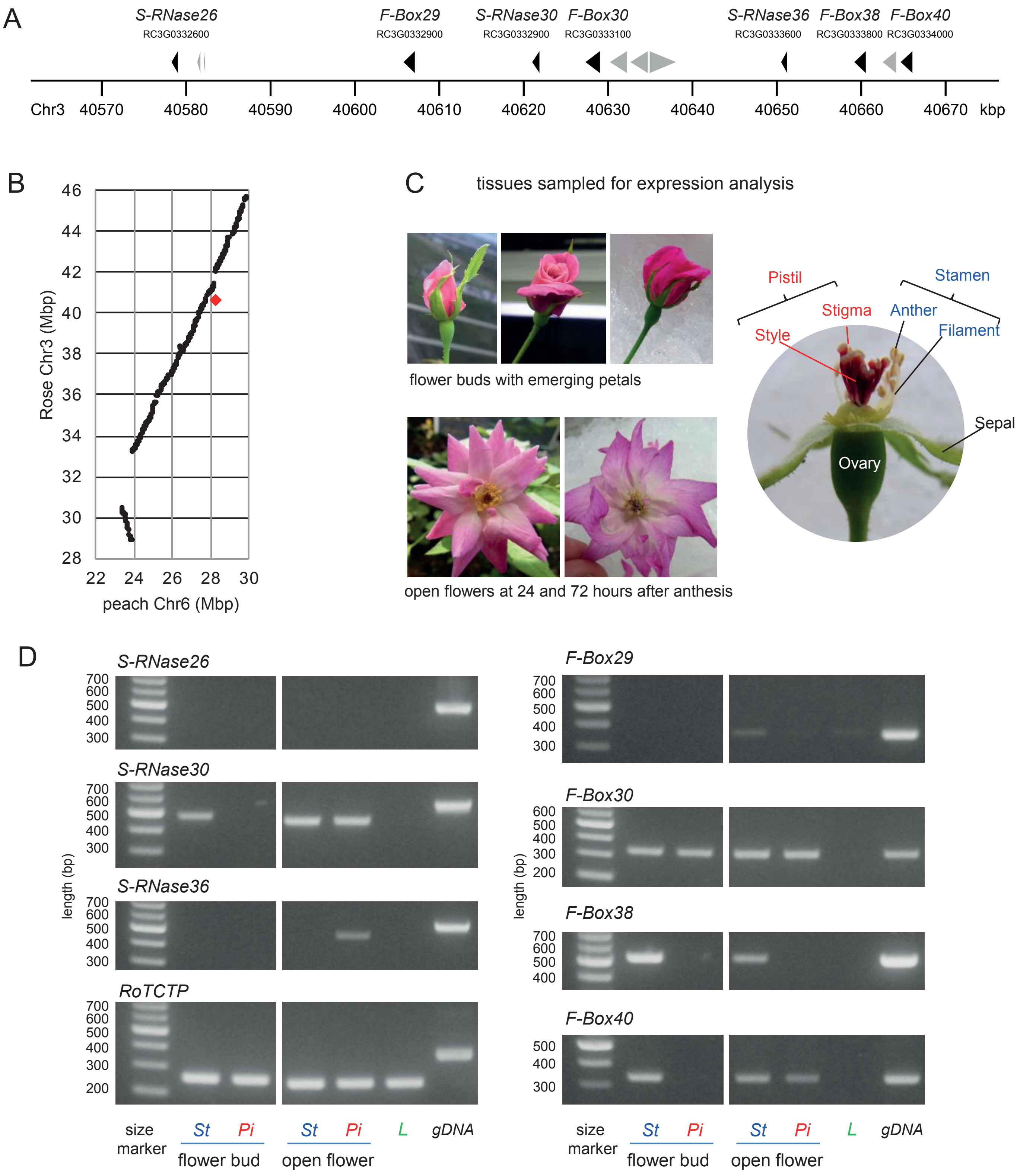

Supplementary Figure 9. Candidate genomic region of the self-incompatibility locus on chromosome 3 (Chr3) of HapOB. A) Location of candidate genes in a 100 kbp region of the rose S-locus; *S-RNase* and *F-Box* genes are depicted as black arrows, other genes as gray arrows. B) synteny between the genomic regions surrounding the S-locus in peach (red diamond, Chr6) and rose (Chr3). C) Tissues from flower bud and open flower sampled for expression analysis by RT-PCR. D) RT-PCR analyses of candidate genes (*S-RNase* and *F-Box*). St for stamen; Pi for pistils; L for leaves. Genomic DNA (gDNA) is used as positive control, and *RoTCTP* is used as house-keeping gene. The flower sampling and RT-PCR analyses were repeated four times independently (two times for one individual plant and two times for an other individual plant) with similar results.

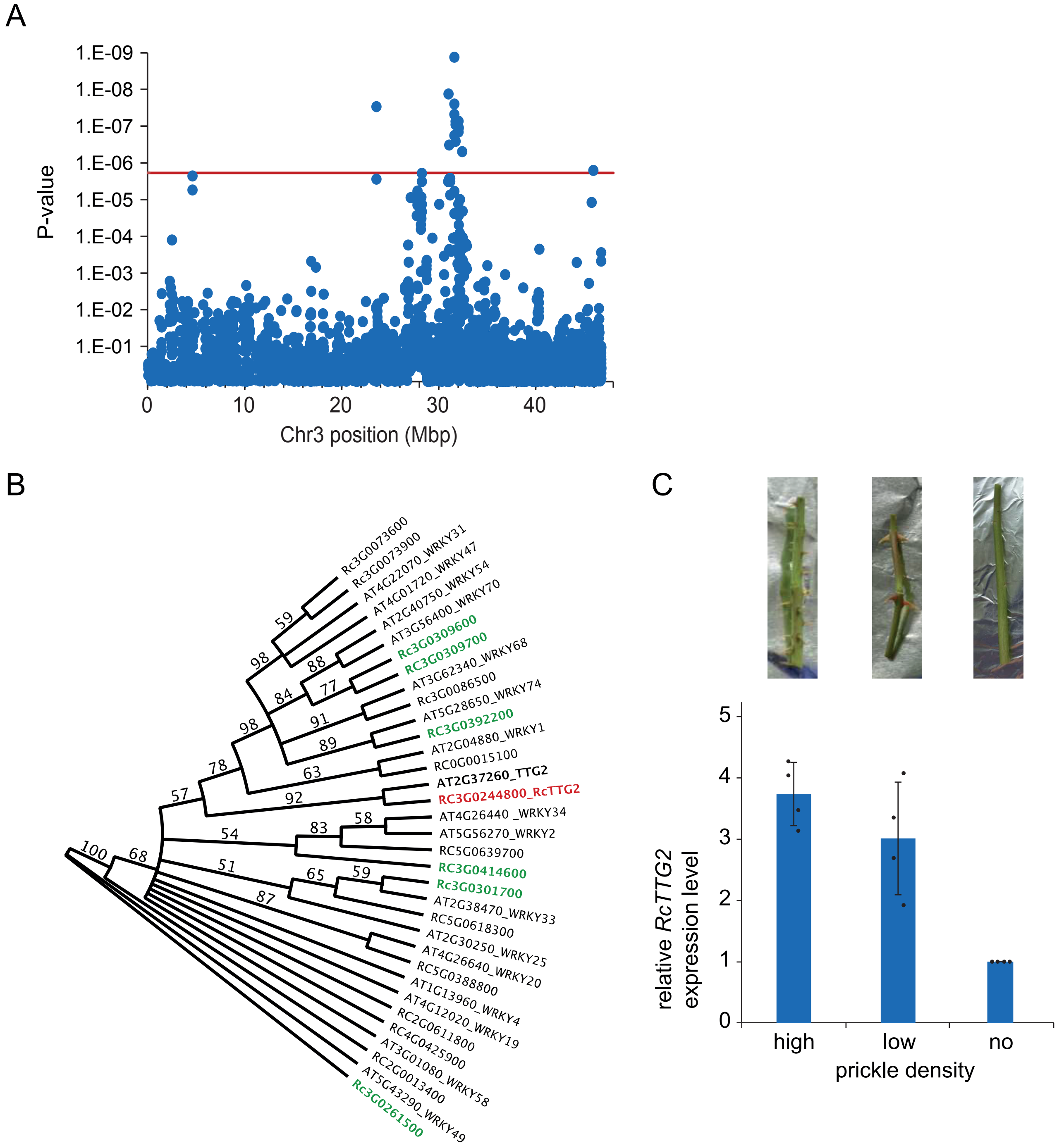

Supplementary Figure 10. A *TTG2* homolog is a candidate gene for the control of prickle density. A) GWAS analysis (GLM with quantitative data) showing p-values of the association between SNPs positioned along Chr3 and the absence or presence of prickles. Significant SNPs are located between positions 31 Mbp and 32.4 Mbp. Horizontal red line shows Bonferroni corrected significance level (1.78E-6). B) Phylogenetic analysis of the *Arabidopsis TTG2* clade of the *WRKY* transcription factor family. The rose WRKY transcription factors located in the prickle density QTL region are shown in green and the closest TTG2 homolog is shown in red. The *Arabidopsis* WRKY proteins are numbered according to TAIR nomenclature (http://www.arabidopsis.org). C) *TTG2* transcript accumulation in three different OW individuals with no prickles, low, and high prickle density. The transcript accumulation level was analysed by qPCR and expressed as a ratio relative to the sample with no prickles (mean ± SD). n = two biologically independent stem samples with two technical replicates each, per prickle density type.

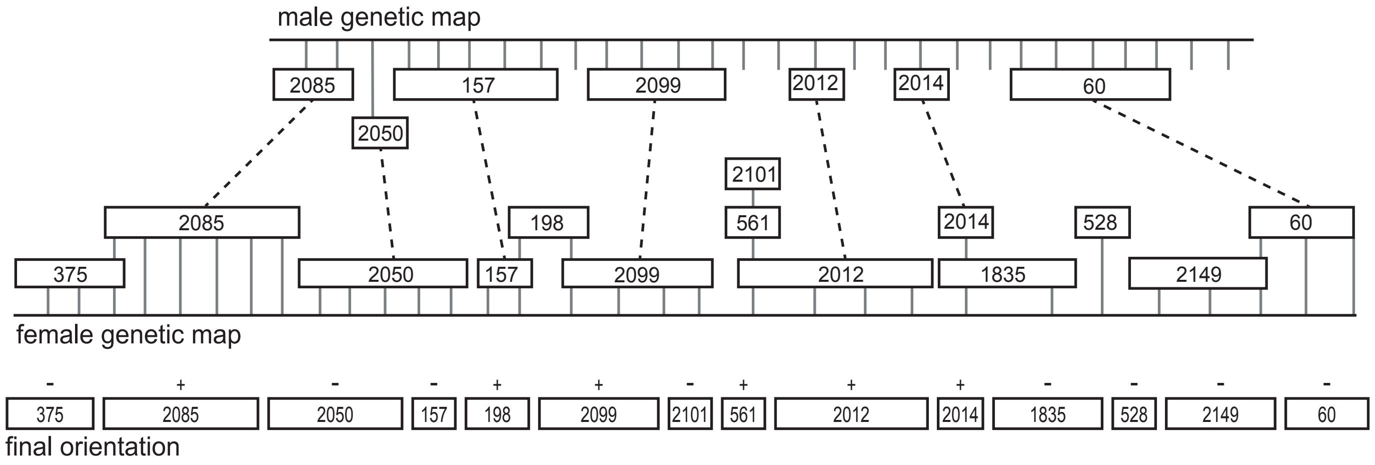

Supplementary Figure 11. Strategy used to position and orient the contigs by anchoring onto the OW genetic map, illustrated on the upper half of Chr5, for the male map (top) and for the female map (bottom). Genetic loci used for anchoring are indicated by gray tick marks (on average 8 SNP markers per locus). Per contig (shown as boxes), sequence orientation is indicated by the + or - sign. Contigs that are only anchored to one locus (e.g. contig 528, 561, 2014 or 2101), are oriented based on synteny with *Fragaria vesca*. Dashed lines connect contigs anchored to the female and male maps. During anchoring, three different cases were encountered. Case 1: The contig is not oriented with the male map, but can be oriented thanks to the female map (e.g. contig 2014, orientation -). Case 2: The contig is anchored only on the male or female map (e.g. contig 375). We manually integrated the contig upstream of contig 2085. Case 3: At a same locus, more than one contig is anchored (e.g. contig 561 and 2101 on the female map). In order to position and orient the contigs, we used synteny with *Fragaria vesca*, and positioned contig 2101 (orientation -) before contig 561 (orientation +), and both between contigs 2099 and 2012.

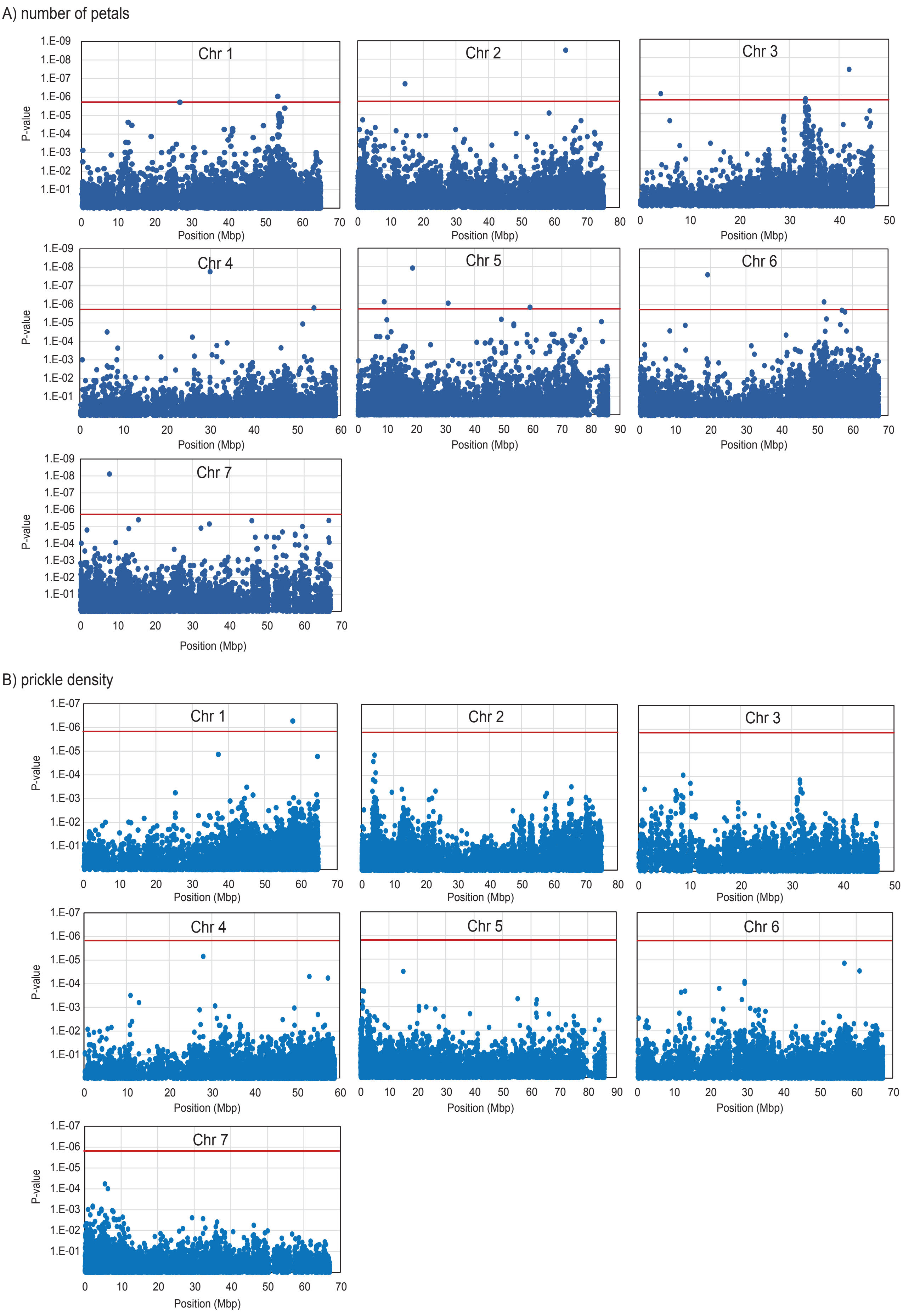

Supplementary Figure 12. GWAS analysis on a panel of 96 rose cultivars for A) number of petals and B) prickle density. The Manhattan plot was calculated with MLM (Q+K model) for all seven chromosomes. For number of petals n = three flowers per cultivar), data were Box-Cox transformed; for prickle density (n = three shoots per cultivar), data were log-transformed before analysis in Tassel. Horizontal red lines show Bonferroni corrected significance level (1.78E-6).
