# Supplementary material for: A high-quality genome sequence of Rosa chinensis to elucidate ornamental traits
Source: Nat Plants. 2018 Jun 11;4(7):473–84. doi: 10.1038/s41477-018-0166-1 (PMC6786968; doi:10.1038/s41477-018-0166-1)
Supplement: Supplementary file 2 — Reporting Summary file [file 41477_2018_166_MOESM2_ESM.pdf]

nature research

Corresponding author(s): Fabrice FOUCHER

Reporting Summary

Nature Research wishes to improve the reproducibility of the work that we publish. This form provides structure for consistency and transparency in reporting. For further information on Nature Research policies, see Authors & Referees and the Editorial Policy Checklist.

Statistical parameters

When statistical analyses are reported, confirm that the following items are present in the relevant location (e.g. figure legend, table legend, main text, or Methods section).

n/a	Confirmed	
☐	☒	The exact sample size (*n*) for each experimental group/condition, given as a discrete number and unit of measurement
☐	☒	An indication of whether measurements were taken from distinct samples or whether the same sample was measured repeatedly
☐	☒	The statistical test(s) used AND whether they are one- or two-sided<br>*Only common tests should be described solely by name; describe more complex techniques in the Methods section.*
☐	☒	A description of all covariates tested
☐	☒	A description of any assumptions or corrections, such as tests of normality and adjustment for multiple comparisons
☐	☒	A full description of the statistics including central tendency (e.g. means) or other basic estimates (e.g. regression coefficient) AND variation (e.g. standard deviation) or associated estimates of uncertainty (e.g. confidence intervals)
☐	☒	For null hypothesis testing, the test statistic (e.g. *F*, *t*, *r*) with confidence intervals, effect sizes, degrees of freedom and *P* value noted<br>*Give P values as exact values whenever suitable.*
☒	☐	For Bayesian analysis, information on the choice of priors and Markov chain Monte Carlo settings
☒	☐	For hierarchical and complex designs, identification of the appropriate level for tests and full reporting of outcomes
☒	☐	Estimates of effect sizes (e.g. Cohen's *d*, Pearson's *r*), indicating how they were calculated
☐	☒	Clearly defined error bars<br>*State explicitly what error bars represent (e.g. SD, SE, CI)*

Our web collection on statistics for biologists may be useful.

Software and code

Policy information about availability of computer code

Data collection	*Provide a description of all commercial, open source and custom code used to collect the data in this study, specifying the version used OR state that no software was used.*
Data analysis	All the softwares used for the assembly and annotation of the genome are described in M&M<br>(name, version, parameters)<br>Genome sequencing<br>* Illumina reads demultiplexing: Casave 1.8<br><br>Genome assembly and anchoring<br>* CANU v1.4, parameters are described, p18)<br>* Illumina read mapping BWA-MEM<br>* Error correction: Pilon<br>* marker anchoring: Blat v.35, BLAST<br>* SPAdes (ver 3.11.1)<br><br>Contamination assessment<br>* MetaGeneAnnotator<br>* contig alignment: BlastX

Genome annotation
* RNA Seq Assembly: SOAPdeno-Trans
* Gene annotation: LTRHarvest, Red, BLASTX, EUGENE
* Transposable Element annotation: REPET package v2.5, TEannot
* Centromer identification: Tandem Repeat Explorer, TAREAN, Repeat Explorer, BLAST, Bowtie, DRAWID

Genetic map construction:
* OW F1 progeny: (Sup M&M p1)
- Genotyping Console and SNPolisher for SNP analysis
- JoinMap v4.0 for genetic map construction

* YW F1 progeny:
- GBS markers: GBSX (v1.1.5), Fast QC, Cutadapt, FASTX-Toolkit 0.0.13, PEAR, BWA 0.7.8, SAMtools 1.2, GATK v3.7,
- JoinMap4.1 and SPSS v2.3 for genetic map construction

* K5 F1 progeny (Sup M&M p3)
- SNP analysis and dosage (FitTetra, PolyMapR)
- MDSMap Package for genetic map construction

GWAS analysis
* Tassel3.0
* kinship matrix calculation: SPAGeDi 1.5
* KASP marker: StepOne Software v2.3

Phylogenetic tree construction using SNP
* GATK Combine Variant and VCF tools to built the SNP alignment
* RAxML v8.1.5 to construct the tree.

Diversity Analysis
* Cutadapt and Faxtx for read quality control
* BWA for SNP identification
* SAMTools and Picard package to remove reads.
* GATK for base quality control and Indel identification
* SnpEff and SnpSift for SNP effects

Synteny analysis
* McSCANX to identify syntenic regions.
* Blast

Custom code :
The R code used for pairwise maximum likelihood recombination and LOD score calculation is available through CRAN (https://CRAN.R-project.org/package=polymapR). The R code used to infer phylogenetic relationship is available under request to the corresponding author.

For manuscripts utilizing custom algorithms or software that are central to the research but not yet described in published literature, software must be made available to editors/reviewers upon request. We strongly encourage code deposition in a community repository (e.g. GitHub). See the Nature Research guidelines for submitting code & software for further information.

Data

Policy information about availability of data

All manuscripts must include a data availability statement. This statement should provide the following information, where applicable:
- Accession codes, unique identifiers, or web links for publicly available datasets
- A list of figures that have associated raw data
- A description of any restrictions on data availability

All the genome data have been made available on a genome browser (https://iris.angers.inra.fr/obh/) and in the public GDR database (https://www.rosaceae.org/species/rosa/chinensis/genome_v1.0) 91. Fasta files of chromosomes and genes (mRNA, Proteins and ncRNA) and gff files for gene models and structural features (TE) can be downloaded on both. Raw data (PacBio and Illumina reads) are available under the following accession (PRJNA445774). RNASeq data used for genome annotation are available under the following SRA accession (SRP128461 for 91/100-5 leaves infected with blackspot and SRP133785 for R. wichurana and 'Yesterday' leaves infected with two powdery mildew pathotypes). Raw data of resequencing of the eight wild Rosa species are available under the SRA accession number SUB3466405

Field-specific reporting

Please select the best fit for your research. If you are not sure, read the appropriate sections before making your selection.

☒ Life sciences ☐ Behavioural & social sciences ☐ Ecological, evolutionary & environmental sciences

For a reference copy of the document with all sections, see nature.com/authors/policies/ReportingSummary-flat.pdf

Life sciences study design

All studies must disclose on these points even when the disclosure is negative.

Sample size	We do not used statistical methods to define the sample size of the progenies. The size of F1 progenies in rose is in the standard for rose genetic maps (151 individuals for OW, 174 for YW and 172 for K5). These sample size (more than 150 individuals) is sufficiant for QTL and major gene detection. Combination of the different progenies allows the delimitation of a small region for cloning the double flower locus. The panel diversity size is 96 individuals (described in Supplementary Table 12), but interesting locus (as the double flower) was validated on a larger panel (238 modern roses)
Data exclusions	For the OW progeny, one individual was excluded for the genetic map construction (too many missing data)
Replication	The replications were done for scoring morphological traits in the F1 progenies and diversity panel:<br>For double flower: described p24 in M&M (for F1 progenies: more than 5 flowers per plants, for GWAS, 3 flowers per plants on 3 clones)<br>For prickle density: on 3 independent shoots (p24 in M&M)<br>For continuous flowering: the scoring was done during 3 years.<br>Replication for qPCR experiments:<br>For each experiment 3 technical and 2 biological replicates were done<br>All attempts at replication were successful.
Randomization	For the diversity panel, the plant were cultivated in a randomised block design with 3 blocks comprising one clone (M&M p19)
Blinding	The data (scoring of morphological traits) were directly collected in the field or in the greenhouse with no blinding. The blinding is not relevant as the genetic analysis is performed after the scoring; therefore the scoring cannot be influenced by expected results.

Reporting for specific materials, systems and methods

Materials & experimental systems

n/a	Involved in the study
☐	☒ Unique biological materials
☒	☐ Antibodies
☒	☐ Eukaryotic cell lines
☒	☐ Palaeontology
☒	☐ Animals and other organisms
☒	☐ Human research participants

Methods

n/a	Involved in the study
☒	☐ ChIP-seq
☐	☒ Flow cytometry
☒	☐ MRI-based neuroimaging

Unique biological materials

Policy information about availability of materials

Obtaining unique materials	All unique materials are readily available from the authors

Flow Cytometry

Plots

Confirm that:

☐ The axis labels state the marker and fluorochrome used (e.g. CD4-FITC).

☐ The axis scales are clearly visible. Include numbers along axes only for bottom left plot of group (a 'group' is an analysis of identical markers).

☐ All plots are contour plots with outliers or pseudocolor plots.

☐ A numerical value for number of cells or percentage (with statistics) is provided.

Methodology

Sample preparation	Callus developed from somatic embryos obtained from anthers was analyzed. The Cystain® absolute PI reagent kit (Sysmex, Germany) was used for sample preparation according to the manufacturer's protocol. The plant material is chopped using a

razor blade. The kit uses Propidium Iodide as a fluorochrome. Leaf material of Solanum lycopersicum 'Stupické polni tyckove rane' was used as an internal standard, with known genome size, and co-chopped with the rose callus.

Instrument

PASIII - equipped with 488 nm 20 mW solid state laser – supplier: Partec (currently Sysmex, Münster, Germany).

Software

Flomax 2.9 – supplier: Quantum Analysis (Münster, Germany)

Cell population abundance

For every analysis between 5,000 and 10,000 nuclei are analyzed. Genome sizes are calculated based on the ratio of the peak position of the rose callus material and the tomato with the known genome size (1916 Mbp/2C) . Three repetitions were performed on different days.

Gating strategy

No gating was applied. The analysis of the plant genome size results in a non-gated one parameter histogram output on a linear scale.

☐ Tick this box to confirm that a figure exemplifying the gating strategy is provided in the Supplementary Information.
